# Evaluating a brief imagery-based intervention for adolescent depression: study protocol for a Phase IIB randomised control trial (INDIGO) in secondary schools

**DOI:** 10.1186/s13063-025-08920-9

**Published:** 2025-07-03

**Authors:** Victoria Pile, Rose Tinch-Taylor, Ben Carter, Jessica Richardson, Lisa De Rijk, Mary Leamy, Simon E. Blackwell, Richard Meiser-Stedman, Barnaby D. Dunn, Sarah Byford, Emily A. Holmes, Cathy Creswell, Patrick Smith

**Affiliations:** 1 Department of Psychology, Institute of Psychiatry, Psychology and Neuroscience, King’s College London, De Crespigny Park, London, SE5 8AF UK; 2 King’s Clinical Trials Unit, Institute of Psychiatry, Psychology and Neuroscience, King’s College London, De Crespigny Park, London, SE5 8AF UK; 3 Department of Biostatistics and Health Informatics, Institute of Psychiatry, Psychology and Neuroscience, King’s College London, De Crespigny Park, London, SE5 8AF UK; 4 Care of Long-term Conditions, Florence Nightingale Faculty of Nursing and Midwifery, King’s College London, London, UK; 5 Department of Clinical Psychology and Experimental Psychopathology, Institute of Psychology, University of Göttingen, Göttingen, Germany; 6 Department of Clinical Psychology and Psychological Therapies, Norwich Medical School, University of East Anglia, Norwich, UK; 7 Mood Disorders Centre, University of Exeter, Exeter, UK; 8 Health Service & Population Research Department, King’s College London, 18 De Crespigny Park, Denmark Hill, London, SE5 8AZ UK; 9 Department of Women’s and Children’s Health, Uppsala University, Uppsala, Sweden; 10 Departments of Psychiatry and Experimental Psychology, University of Oxford, Anna Watts Building, Radcliffe Observatory Quarter, Oxford, OX2 6GG UK

**Keywords:** Depression, Adolescence, Mental imagery, Autobiographical memory, Early intervention, Psychological therapy, School-based intervention, Imagery rescripting, Memory specificity training

## Abstract

**Background:**

There is an urgent need for psychological interventions that can target depression in late adolescence and prevent it from having lifelong implications. Schools have been identified as a promising setting to enhance access to interventions and offer support earlier. We have co-developed a novel intervention, IMAGINE, that targets key cognitive mechanisms implicated in depression across the lifespan. Depression has been associated with distressing negative mental images, a deficit in positive future images and overgeneral autobiographical memories. Interventions targeting these factors have shown clinical promise in adults. Here, we combine techniques targeting these cognitive processes into a novel, brief psychological intervention for adolescent depression. This Phase IIb randomised controlled trial will evaluate IMAGINE compared to an active psychological intervention.

**Methods/design:**

One hundred sixty adolescents (aged 16–18) with high levels of depressive symptoms will be recruited from schools. Participants will be randomly allocated to IMAGINE or the active psychological control intervention, non-directive support (NDS). Assessment will take place at baseline, 8-, 16- and 24-week post randomisation. The primary objective is to establish whether IMAGINE reduces symptoms of depression, relative to NDS, at 8 weeks following randomisation. Secondary objectives include whether changes in depression are maintained at 16- and 24-week follow-up, the efficacy of IMAGINE on secondary clinical outcomes and key cognitive mechanisms and, finally, to assess outcomes around acceptability, safety and adherence.

**Discussion:**

If IMAGINE is shown to be safe and clinically effective, an effectiveness-implementation hybrid RCT will be indicated. If rolled out as an intervention, IMAGINE would significantly extend the range of effective therapies available for adolescent depression.

**Trial registration:**

ISRCTN, ISRCTN14015295. Registered 11 September 2023, https://doi.org/10.1186/ISRCTN14015295.

**Supplementary Information:**

The online version contains supplementary material available at 10.1186/s13063-025-08920-9.

## Introduction

Depression is commonly experienced in late adolescence. Significantly impairing symptoms of depression are present in 5% of 17–19-year-olds [1]. The peak age of first onset of depression is in adolescence, with onset during adolescence (rather than adulthood) associated with more recurrences and an increased risk of chronicity [2, 3]. Depression in late adolescence is associated with higher social dysfunction, poorer academic performance, more physical ill health complaints, and more completed suicides [4–6]. Indeed, of all the mental health disorders emerging in youth, depression impacts the most on health throughout the lifetime (in terms of Years Lost to Disability) [7]. There is an urgent need for early interventions to prevent depression becoming entrenched and reduce lifelong distress and disability. Yet, in the UK, 75% of adolescents with depression do not receive an intervention [8]; gold-standard interventions require several months of sessions with experienced therapists, and current evidence-based psychotherapies for youth to show only a modest advantage over usual care, if any [9, 10]. Indeed, a recent meta-analysis [11] suggested that more than 60% of youth receiving therapy for depression do not respond. In addition, adolescents with lived experience and psychological therapists identify a need to have a greater range of evidence-based interventions for depression, for example to enable informed choice [12]. One approach to improve access and identify adolescents earlier is to deliver interventions in school settings [13–15]. Developing protocol-driven interventions that target specific cognitive mechanisms may allow us to enhance efficacy and improve fidelity when delivering interventions across a broader range of therapists. Here, we evaluate a novel early intervention for adolescent depression (IMAGINE) which targets cognitive factors implicated in depression: dysfunctional mental imagery and maladaptive memory processes. IMAGINE has been designed for young people aged 16 to 18 (which falls in the period of “late adolescence” [16]) as this represents a period of vulnerability to depression but also opportunity to prevent cognitive styles becoming entrenched. Depressive symptoms are very common and there is evidence that rates of difficulties are continuing to rise for this age group (in contrast to younger age groups) [1, 17]. Yet, it is also a period characterised by increased flexibility, learning potential and independence [18].

Two promising targets for interventions are mental imagery and autobiographical memory specificity. These targets are suggested from evidence that people with depression frequently experience past distressing images, struggle to imagine positive events in their future and do not recall memories in detail [11–15]. Our mental experience includes verbal thoughts as well as mental imagery, which involves the ability to simulate and manipulate multisensory experiences within the so-called “mind’s eye” using internal representations (e.g. memories of past events and projections of future events) [19]. Mental images can range from distressing memories of past events, entering awareness unbidden and producing strong psychophysiological stress responses (i.e. intrusions), to images of possible future events deliberately brought to mind (e.g. when planning) [20]. Across the lifespan, there is evidence that depression is associated with an increased frequency of distressing intrusive images as well as a deficit in generating positive future imagery [21–26]. Mental imagery can be a treatment target (e.g. intrusions) as well as a therapeutic vehicle to enhance interventions. For example, imagery rescripting uses imagery techniques to target intrusions [27] and recent work generates and enhances motivational imagery [28]. Reviews of psychological interventions that harness imagery approaches have identified it as a promising approach for reducing symptoms of depression and highlighted that it has transdiagnostic effects (e.g. on anxiety and self-esteem) [19]. Imagery interventions could be particularly suited to this period of cognitive development, with late adolescence representing a balance between heightened reliance on image-based processing (relative to adults) [29] and greater degree of cognitive control over emotional mental imagery (relative to children).

Having less specific autobiographical memories (or overgeneral memory, OGM) has been consistently implicated in adolescent depression, being not only associated with current symptoms but also with the onset, maintenance and relapse of depression [30]. Difficulties in accessing and processing specific autobiographical memory has implications for daily cognitive functioning, including planning, problem-solving and social interaction [25] as well as difficulties imagining future events [31]. Importantly, research with adults has demonstrated that OGM does not reflect a general deficit in memory functioning [24] and is not purely a correlate of current low mood [32]. Researchers suggest that overgeneral memory may result from an individual avoiding the specific details of negative events (to reduce its emotional intensity) but this in turn reduces specificity across all memories [24]. Early-stage trials in adolescents and adults indicate that a simple approach to increase memory specificity (generating specific memories to cue words e.g. happy) can reduce depressive symptomatology and improve day-to-day cognition [33–35]. Further to enhancing specificity, the ability to alternate between specific and general memories has been associated with symptoms of depression [36]. Incorporating techniques to encourage flexibility between categories of autobiographical memory could, therefore, be valuable [37].

### Development and initial evaluation of IMAGINE

IMAGINE (Integrating Memories And Generating Images of New Experiences) has been developed following MRC guidelines [38]. Co-design has included consulting adolescents and adults with lived experience, parents of adolescents with lived experience, teachers and clinicians (for example [27]). IMAGINE uses three techniques: (1) imagery rescripting to process distressing intrusive memories; (2) prospective imagery generation to improve impoverished positive imagery and (3) memory specificity/flexibility training to enhance specificity and access to memories (and their associated values).

IMAGINE has been initially tested in a case series and feasibility Randomised Controlled Trial (RCT) [39, 40]. In the RCT, the feasibility, acceptability and safety findings exceeded the pre-defined continuation criteria: recruitment completed within 11 months; 89% retention; high acceptability; and no harm indicated. Clinical promise was also indicated for IMAGINE compared to an active intervention for depression (non-directive support, NDS): large effects in favour of IMAGINE at postintervention in reducing symptoms of depression (*d* = 1.34, 95% CI [0.80, 1.87]), which were maintained 3 months later (*d* = 0.96, 95% CI [0.33, 1.59]). Data also indicated improvements in anxiety (*d* = 0.51) and cognitive targets (e.g. memory specificity, *d* = 0.79 and vividness of positive future imagery, *d* = 0.44), compared to NDS. In the process evaluation [41], the active ingredients identified by participants were consistent with (and extended our understanding of) the theoretical basis of the intervention. The four themes were (1) processing negative experiences and letting go (e.g. “Opening up about my secondary school experiences, I felt like I was able to relieve such emotions and…deal with such negative thoughts”) (2) imagining positive future events (e.g. “Imagining myself in that position was really eye-opening and…now I’m motivated to work harder to see myself in that position”); (3) understanding how memory works and being able to remember memories in more detail (e.g. “I saw my memories in a different way, I see it in a more cinematic way, I suppose, yeah, like I choose to open them and close them.”); (4) understanding and being kinder to myself (e.g. I didn’t have clear memories, I felt like I can’t express myself…I didn’t know what words to use or what words would be right. Because of the groupings, of when we group the words, and then the clear memory, I can express myself more”).

### Current study

We now aim to test IMAGINE in a Phase IIb RCT (INDIGO, INterventions for Depression In younG peOple), with the interventions delivered by graduate psychological therapists in schools/colleges. The control intervention will be non-directive support (NDS), which aims to control for the non-specific aspects of therapy (e.g. speaking with an empathic therapist) that could contribute to symptom reduction {6b}. NDS is a NICE recommended intervention for mild depression in adolescents [42].

In addition to assessing whether IMAGINE reduces symptoms of depression, it is valuable to understand the extent to which IMAGINE (vs. NDS) improves school and social impairment as well as related difficulties (e.g. anxiety, low self-worth, insomnia) and whether there are changes in key cognitive mechanisms (e.g. emotional mental imagery, memory specificity, self-compassion, activity levels and rumination) following the interventions. Understanding which related difficulties are reduced could, for example, enable better tailoring of the intervention to an individual’s presenting difficulties and improved informed choice for this population. Furthermore, insight into the cognitive mechanisms is important for retaining effect sizes when scaling interventions, explaining how interventions work and developing therapist training.

#### Objectives {7}

1. The trial’s primary objective is to evaluate whether, in adolescents aged 16–18, a brief imagery-based intervention (IMAGINE) reduces symptoms of depression relative to an active control intervention (non-directive support; NDS) at 8 weeks following randomisation.
2. Our secondary objectives are to:
  2.1 Establish whether any observed changes in symptoms of depression are maintained at 16- and 24-week follow-up.
  2.2 Establish the efficacy of IMAGINE (compared to an active control) on secondary clinical outcomes. This includes changes in anxiety, self-worth, sleep difficulties, school and social impairment and symptoms of intrusions and avoidance to a negative event.
  2.3 Establish whether there are changes in the key cognitive mechanisms (e.g. mental imagery and memory specificity) and associated mechanisms (self-compassion, activity levels, rumination) when receiving IMAGINE, relative to NDS.
  2.4 To further assess (a) acceptability of the interventions to participants; (b) safety of the interventions; (c) adherence to the intervention protocol by clinicians and participants, fidelity of the intervention and contamination (both therapist and peer to peer contamination).

To prepare for a future pragmatic effectiveness-implementation hybrid RCT (e.g. a multi-centre design with the training incorporated into pre-existing training pathways), we also have exploratory objectives to (1) provide the groundwork for the health economics component of a future trial and (2) to use a process evaluation to explore factors impacting the methodology and delivery of INDIGO. The process evaluation is an embedded qualitative study to which participants will be invited following completion of the trial. These findings will be reported outside of the primary results paper.

#### Trial design and timeline {8, 13}

**Fig. 1:**
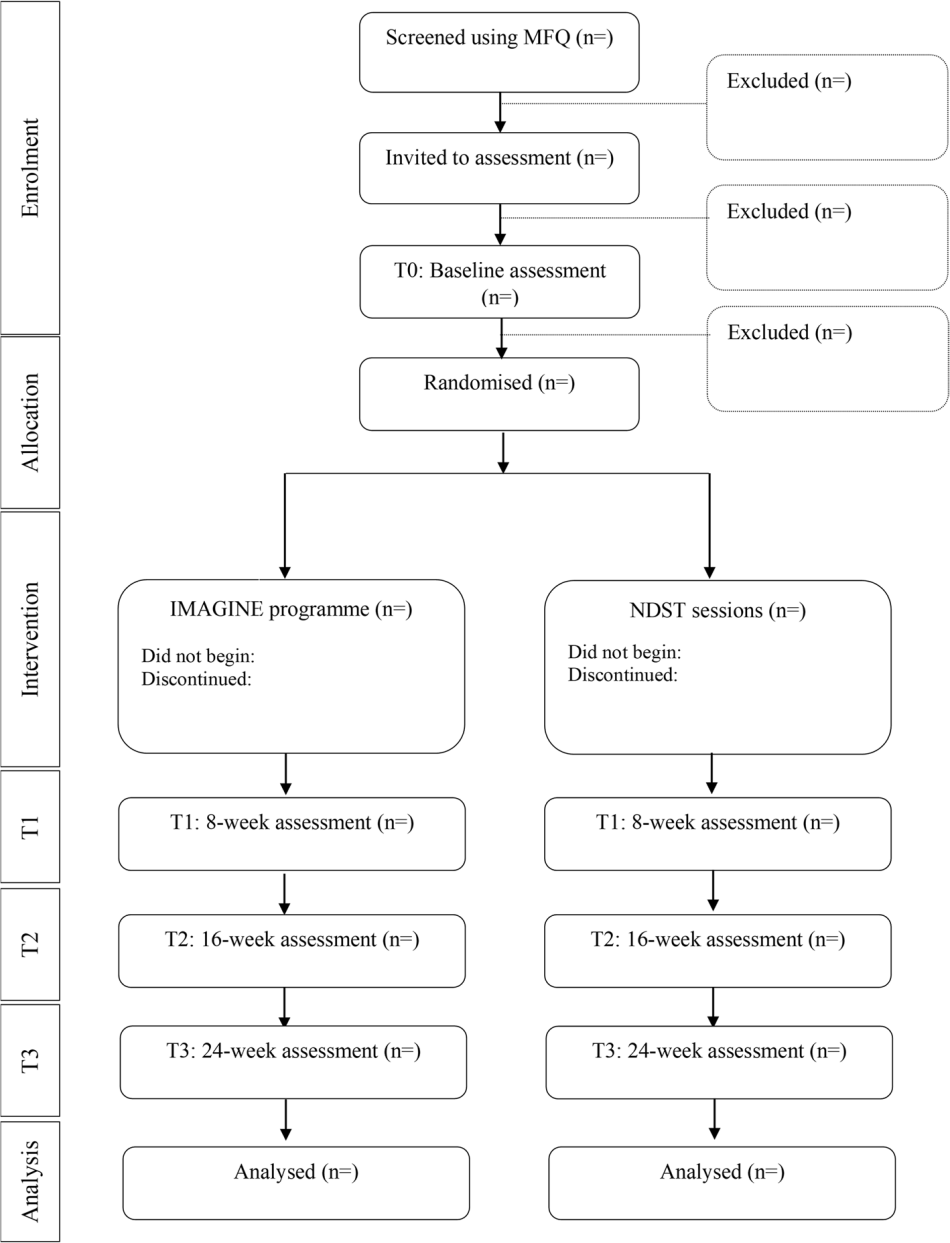
Flow through trial in CONSORT diagram

This is a multi-school, assessor blinded, parallel group, superiority RCT in adolescents in England comparing IMAGINE versus active control (NDS) with a 1:1 allocation ratio. Eligible participants will be randomly allocated to receive IMAGINE or NDS. All participants will receive an active intervention, and both interventions aim to improve mood and self-esteem. These methods are based on the INDIGO trial protocol (version 1; 11th September 2023), approved by the Trial Steering Committee and Trial Management Group. This protocol follows the SPIRIT (Standard Protocol Items: Recommendations for Interventional Trials) reporting guidance (please see supplementary materials for SPIRIT checklist) with curly brackets used to identify corresponding items on the checklist. Please see Table 1 for the participant timeline and Fig. 1 for the CONSORT diagram.

**Table 1:**
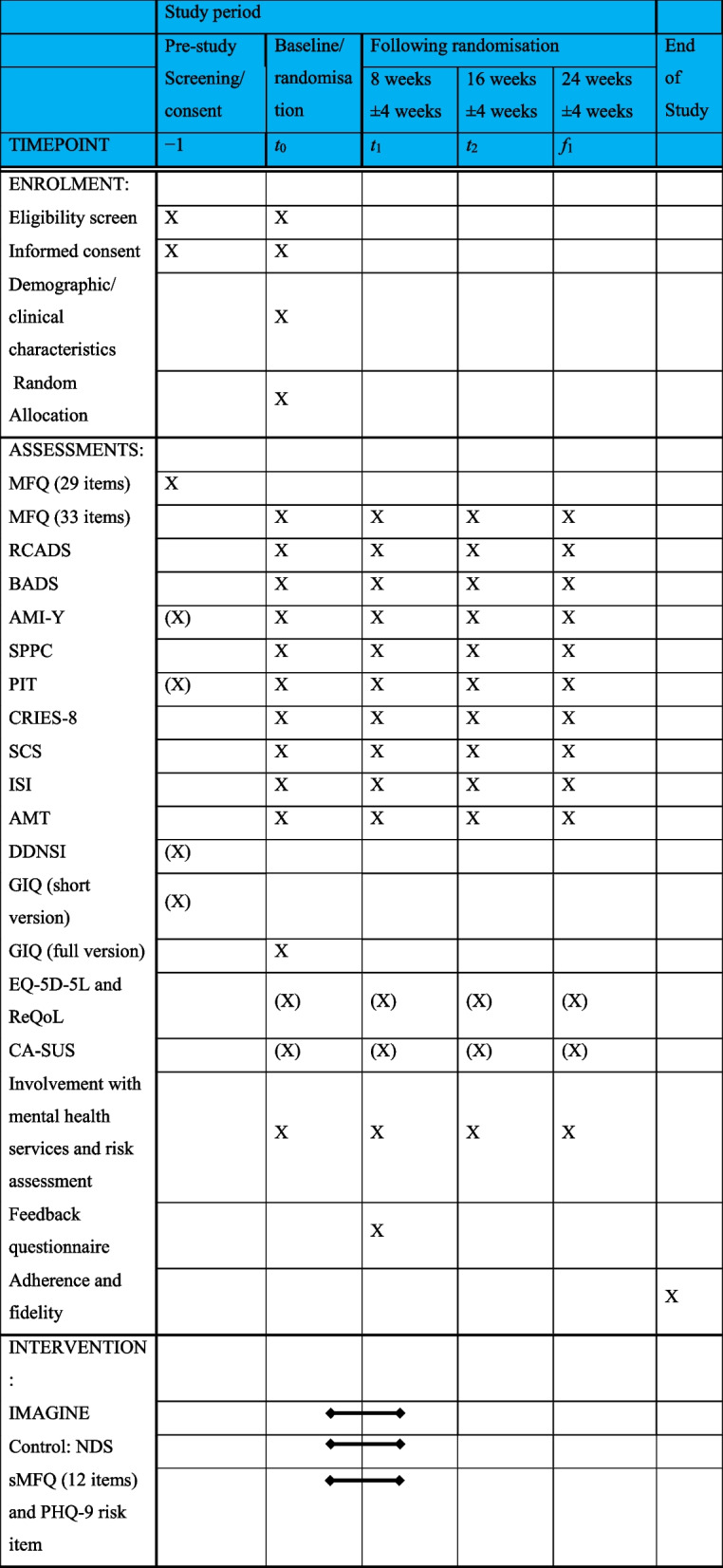
Participant timeline

(X) not included in primary paper

There are two stages to the project: the screening stage (stage 1) and the intervention stage (stage 2). In stage 1, pupils aged 16–18 will be asked to complete the Mood and Feelings Questionnaire (MFQ, at screening (only) the four risk items will be removed from the MFQ due to ethical considerations). Those scoring above clinical cut-off will be invited to stage 2.

In stage 2, assessments will be at baseline (prior to randomisation), 8-, 16- and 24-week following randomisation. Demographic factors will be measured at baseline and inclusion/exclusion criteria checked. Each assessment will consist of a battery of questionnaires (the majority standardised measures), the autobiographical memory task, a semi-structured clinical interview (to assess risk and history of mental health difficulties) and a semi-structured interview to collect service use data. Each assess79ment will be approximately 1 h long, and the questionnaires will be split into essential questionnaires and optional questionnaires.

#### Health economics

Data will be collected to examine the comprehensiveness, acceptability and feasibility of economic measures of service use, used to estimate costs, and health-related quality of life, used to estimate quality-adjusted life years (QALYs), for application in a future effectiveness-implementation hybrid RCT. Given that the current study aims to assess efficacy (rather than effectiveness) and the intervention, and its cost, may differ in routine NHS practice, a full definitive analysis of cost effectiveness is not appropriate. However, economic data (e.g. service use and QALYs) will be collected to provide some initial insights to guide a future trial.

#### Embedded process evaluation

The process evaluation will consist of (1) Feedback on the interventions: participants will complete a feedback form at 8-week following randomisation. This will include open-ended questions about the intervention; (2) a subset of trial participants will be invited to take part in semi-structured interviews following completion of their participation in the trial; (3) the graduate psychological therapists who deliver IMAGINE and NDS will be invited to take part in semi-structured interviews following competition of their role. The interview schedules will be developed with Patient and Public Involvement (PPI) input. Interview data will be audio-recorded and analysed using thematic analysis, applying a framework approach [43]. These interviews will be conducted, analysed and reported separately from the main trial.

A previous process evaluation [41] has explored the active ingredients and valued outcomes of IMAGINE. The current process evaluation will aim to expand on these previous findings consistent with guidelines [38], for example by further exploring the factors impacting implementation, mechanisms of change and contextual factors. The aims for the semi-structured interviews are:

Therapist interviews:

- To understand whether there are some adolescents who are more likely to benefit from IMAGINE and some more likely to benefit from NDS.
- Explore the factors that supported or inhibited the effective delivery of IMAGINE and NDS in the INDIGO trial.
- To understand possible impacts on the psychological therapists delivering IMAGINE and/or NDS.

Participant interviews:

- To explore how mental well-being and low mood is conceptualised by indicated sample (scoring above clinical cut-off for depression) of adolescents and how this has changed over their life so far (i.e. have they had periods of recovery).
- To investigate the interaction between mental well-being and their ability to imagine the past and future.
- To further understand the interaction between changes in mental health and access to help and support (INDIGO interventions, other forms of support) in this group.

## Methods: participants and interventions

### Study population {10}

All participants will be recruited from secondary schools and colleges in Greater London, United Kingdom.

#### Inclusion criteria

1. Aged 16–18.
2. Able to provide informed consent.
3. Willing and able to engage in psychological intervention and complete assessments.
4. Scoring above clinical cut-off on Mood and Feelings Questionnaire at both screen and baseline assessment. A clinical cut-off of 20 at baseline assessment with the 33 item MFQ and a score of 17 at screen on the 29 item MFQ (a score of 20 on the 33 item MFQ was chosen based on previous literature [44–46] and a score of 17 chosen to account for the removal of the risk items at screening).

### Exclusion criteria

1. Diagnosis of learning disability or significant head injury, neurological disorder or epilepsy.
2. Unable to fluently communicate in spoken English.
3. Currently receiving another psychological intervention (including school counselling).
4. Moderate to high levels of risk. This will be verbally assessed with the participant at first interview and discussed in supervision with the chief investigator (VP). This will be based on clinical judgement, an outline is that Imagery Rescripting in this context is unlikely to be appropriate for participants presenting with current and/or significant self-harm (e.g. which requires medical attention); active suicidal ideation and an active plan to harm themselves; and/or those presenting with significant risk to others. The clinical decision will be informed by their history of risk.
5. Current diagnosis of bipolar disorder, post-traumatic stress disorder (PTSD) or psychosis. This will be stated in the information sheet and the participant will be asked at first assessment whether they have been given a diagnosis and by whom.
6. Other significant conditions or factors that contraindicate the individual’s participation in the trial. These conditions or factors will be carefully recorded to further operationalise them in a future implementation trial.

### Study setting {9}

The setting for this study will be secondary schools and sixth form colleges across Greater London. (The intervention may also take place at the Institute of Psychiatry, Psychology and Neuroscience or virtually for a minority of participants who are not able to participate at their school/college).

### Interventions {11a and 6b}

Both interventions will consist of three to four individual sessions. Sessions will last up to a maximum of 90 min, with the possibility of participants taking breaks during the session if needed. The project team will ask the participant for their school timetable and will schedule the meetings at times when they do not have lessons.

It is anticipated that all sessions will take place in a private room in the school or college. However, there are exceptions including (1) the sessions taking place at the Institute of Psychiatry, Psychology and Neuroscience if there are difficulties with completing the session at school or college (for example a young person expresses concerns about taking part in the school environment); and (2) the sessions taking place remotely if (a) the young person states a clear preference for remote delivery and it is possible within the safeguarding procedures of the school or (b) if school/government policy or advice means that external practitioners are unable to enter school premises.

#### Experimental intervention: IMAGINE (Integrating Memories And Generating New Experiences)

The intervention will follow a treatment manual will be accompanied by an intervention workbook and a mobile phone application for homework delivery. The intervention will combine (A) imagery protocols to reduce the distress associated with negative images and build positive future images and (B) Memory Specificity Training with values work to increase specificity and access to memories. Please see Table 2 for a description of these techniques. The protocols have been co-developed with young people and adults with lived experience, parents and clinicians (for example [27]). The intervention includes:

1. Rationale for “training memories”, including the role of memories, the encapsulated meaning of memories and relationship between memories, mood and behaviour.
2. Imagery Rescripting for a negative past image that is associated with school (e.g. a bullying experience in school). The procedure follows three steps, (1) “Reliving” (2) “Mastery” (3) “Compassion”, recalling the image in a different way in each step. The protocol was adapted for adolescents based on previous adult literature [47–50].
3. Scripting positive future imagery (e.g. graduating from university) using a three-stage protocol: (1) “‘Imagining”, (2) “Looking back” (3) “Moving forwards”. The procedure was developed based on experimental literature [51] and literature on positive image generation [49, 52, 53].
4. Rationale and practice for making memories more specific and detailed. This will include in session exercises and between session tasks delivered by a mobile phone application. A values-based exercise will highlight links between specific memories and more general value-based categories.

**Table 2:** Therapeutic techniques

**Technique**	**Procedure**
Imagery rescripting for a negative past event	1) Reliving: Relive image at the age of the participant when the event took place, provide specific details. The meaning and significance of this image is discussed. 2) Mastery: Identify a kind, compassionate other. Relive image as compassionate other who can intervene. 3) Compassion: Relive image as themselves with intervention from step 2.
*Example*	*Freezing and not writing anything in an exam (associated meaning: I'm a failure; I have let my parents down). *
Scripting positive future imagery	(1) Imagining: Generate vivid positive future image, specific and rich in sensory detail. (2) Looking back: Imagine self as having achieved goal (future self), identify positive and difficult elements, coping and resilience strategies. (3) Moving forwards: Imagine progressing from current to future self.
*Example*	*Success in their chosen career (being in an art studio surrounded by their work).*
Memory specificity training with values work	Receive cue word (e.g. music) each day; respond with a specific memory. In session 4, generated memories (from the app and sessions) are reviewed and related to their values.
*Example*	*Specific Memory: Dancing with Dad after maths exam.* *Value: supportive family relationships. *

Throughout the imagery exercises, participants are asked to generate as much detail as possible (including sensory information) as well as thoughts, feelings and the meaning of the images to them. Both imagery rescripting and scripting positive future imagery include techniques aiming to enhance self-compassion and perspective taking. Homework tasks are delivered via a mobile phone application which the participants download onto their phones and prompts them to complete a task each day for 3 weeks (i.e. between sessions 1 and 4).

#### Control intervention: non-directive support

The therapist team will deliver “non-directive support” (NDS). NDS involves planned delivery of individual sessions with an empathic, concerned professional for emotional support, non-directive problem solving and monitoring (e.g. depressive symptoms and school attendance). NDS is based on the principles of non-directive supportive therapy and will follow a treatment guide. This intervention is designed to control for factors that, other than active components of therapy, could contribute to change such as passage of time and non-specific aspects of therapy (e.g. speaking to an empathic therapist). NDS is a variant of counselling, recommended by NICE [42] as an intervention for depression in children and young people. Here, it will be matched for contact time and frequency of sessions.

#### Intervention provider

The majority of intervention sessions will be delivered by research assistants attached to the trial. These research assistants will be graduate psychological therapists (e.g. person with NHS England approved Postgraduate Diploma in Evidence-based Low-intensity Psychological Treatments or graduates in psychology with previous therapeutic experience). The therapists will deliver both interventions. In the event that we require additional psychological therapists (e.g. to meet the intended sample size or if a therapist becomes unavailable after beginning the intervention) contingency plans include (1) recruiting and training other psychological therapists who are pre-qualification (e.g. those completing the doctorate in Clinical Psychology or those completing a PhD in a related area) and (2) qualified Clinical Psychologists to deliver the interventions.

Contamination between the arms will be minimised through expert training and supervision (including bringing tapes to supervision), clear protocols and components for each intervention and will be monitored through adherence checks (independent ratings of session tapes and questions on the feedback questionnaire).

#### Training and supervision for therapists in IMAGINE and NDS

Qualitative interviews were conducted with 10 psychological graduate psychological therapists to understand their training needs. This indicated a 2-day workshop (including presentations, role play, break-out groups, audio examples of the interventions) and weekly supervision (including reviewing recordings) would be appropriate. This training will be provided separately for NDS and IMAGINE and all therapists will be trained in both approaches. Expert supervision will be provided separately for IMAGINE and NDS.

### Criteria for discontinuing or modifying intervention for a given participant {11b}

The content of both the experimental and control interventions are designed to be tailored to the needs of the individual participant, whilst remaining consistent with the intervention manuals. The interventions will be discontinued for a given trial participant if a serious adverse event is reported that is deemed to be associated with the intervention by the chief investigator, supervisors and/or Independent Chair of the TSC. There will be a risk question included in the short form of the MFQ at each intervention session. If this item is endorsed, the therapist will ask follow-up questions. The clinical interview at each assessment session will probe for any adverse events since the previous assessment.

#### Therapist adherence {11c} and equivalence of intervention delivery

Sessions will be audio recorded if the young person consents. A small sample of these recordings will be rated for adherence to the intervention manuals, competence and intervention differentiation by a psychologist, who is not part of the trial team. They will be rated against a modified version of the cognitive therapy scale [54]; this scale was modified for the feasibility RCT to capture elements of the interventions that are unique to each one.

Allegiance bias will be minimised by having separate training and supervision for both arms. The following will provide insight into how comparable the therapies are and as an indicator of allegiance bias: (1) the Credibility/Expectancy Questionnaire [55]; (2) the feedback questionnaire at the 8-week assessment (which asks questions assessing the therapeutic relationship) and (3) independent ratings of competence and treatment differentiation.

### Study adherence and participant compliance {11c}

The key indicator of adherence will be attendance at planned sessions. For a participant in either intervention group to be considered as adhering to the intervention, 3 or more intervention sessions must be completed. To enhance participant adherence to the intervention, strategies will be put in place to remind participants of planned sessions, such as message reminders. Both the therapist and participant will also make a note of agreed homework tasks. Compliance with homework tasks (i.e. number of tasks completed will also be monitored).

### Concomitant care during and after the trial {11d and 30}

Concurrent psychological therapy is an exclusion criterion for entry into the trial. However, it is possible that participants begin alternative interventions after eligibility criteria are checked. Psychological therapies and medication for mental health will be recorded and reported per intervention group. Specific post-trial care is not offered but, where clinically indicated and consent is given, participants will be signposted to relevant services.

#### Intervention records

The psychological therapist will keep notes of each session, which will record key details such as length of session and exercises completed. With participants’ consent, all intervention sessions will be audio-recorded for the purpose of supervision and fidelity checks of adherence to treatment condition. However, eligible participants will not be excluded from the trial if they do not consent to sessions being audiotaped. Audio-recordings will be anonymised and stored digitally on a password-protected computer.

### Methods: outcomes {12} and data collection plan {18a}

All questionnaire measures, the Autobiographical Memory Task and the clinical interviews will be administered at four time points: baseline, 8, 16 and 24 weeks after randomisation. The questionnaires will be split into two sets: (a) essential questionnaires (general information questionnaire, MFQ, RCADS, BADS) and (b) optional questionnaires (EQ-5D-5, ReQoL, AMI-Y, SPPC, PIT, CRIES, SCS, ISI). Please see the Statistical Analysis Plan (supplementary materials) for more information.

#### Primary outcome

The primary outcome is depression symptoms using the Mood and Feelings Questionnaire (long version, MFQ) at 8 weeks following randomisation. The MFQ is a self-report measure of depression. The MFQ is a well-validated and widely used measure of depression in NHS and research settings. It demonstrated excellent internal consistency, high test re-test reliability as well as concurrent and convergent validity [57]. The child self-report version will be used [58].

#### Secondary outcomes

To answer our secondary objectives, the following measures will be collected.

##### Objective 2.1: Depression symptoms at follow-up

Depression symptoms, 16 and 24 weeks after randomisation, will be measured using the MFQ (described above).

##### Objectives 2.2 and 2.3: Secondary clinical outcomes and cognitive mechanisms

- The anxiety subscales from the Revised Children’s Anxiety and Depression scale (RCADs; [59] will be administered to measure anxiety. This is a youth self-report questionnaire. All the anxiety subscales will be administered: separation anxiety disorder, social phobia, generalised anxiety disorder, panic disorder and obsessive-compulsive disorder. This provides a total Anxiety Scale (sum of 5 anxiety subscales) and individual subscale scores. It has been shown to have good internal consistency, test-re-test reliability and concurrent and convergent validity [59–61].
- The Self-worth subscale of the Harter Self-Perception Scale (SPPC [62] will be administered to measure general self-worth. The Harter Self-Perception Scale is a self-report questionnaire with five subscales but only the self-worth subscale will be administered. The self-worth subscale consists of five items. The Harter Self-Perception Scale has shown good internal consistency and concurrent and construct validity [63, 64].
- The Insomnia Severity Index [65] will be administered to measure insomnia. The scale consists of seven items rating insomnia. There is one version used across this age range and has been shown to be valid and reliable in youth [66]
- The Behavioural Activation for Depression Scale (BADS; [67]) will measure school and social impairment as well as activation and avoidance/rumination. The BADS is a 25-item questionnaire comprising four subscales: Activation; Avoidance/Rumination; School Impairment; and Social Impairment. The BADS has been shown to have good internal consistency, test-retest reliability and construct validity [67].
- The Child Revised Impact of Event Scale (CRIES; [68]) will be administered to measure symptoms of intrusions and avoidance in reference to a negative event. The eight-item version of the CRIES will be used. The CRIES has been shown to have good internal consistency, test-retest reliability and discriminant and convergent validity [69–71].
- The Assessment of Mental Imagery in Youth (AMI-Y) will assess responses to emotional mental imagery. It is a questionnaire measure co-developed with adolescents for this trial and the psychometrics have not yet been evaluated. It has been developed based on the process evaluation for IMAGINE [72] and the Imagery Interview[73]. There are two scales, one where participants rate a positive image and one where they rate a negative image.
- The Prospective Imagery Task (PIT, based on [74, 75]; adapted for use in adolescence [76]) will measure vividness of prospective emotional mental imagery. The scale includes seven negative and seven positive scenarios and there are, therefore, two subscales (negative imagery and positive imagery). The PIT has demonstrated very good structural validity and convergent validity with mixed findings on reliability [76, 77]
- The Autobiographical Memory Task [AMT [78] will be administered to measure memory specificity following the original procedure and coding scheme [78]. Participants will be provided with an explanation and examples. They will be asked to give a specific memory to ten cue words (five positive; five negative). Participants will be given 60 seconds to respond to each cue word. When consent is given, the AMT will be audio-recorded and the responses co-rated. Responses are coded as specific, general categoric, general extended, semantic association or omission. Excellent inter-rater consistency was obtained in the feasibility RCT [39].
- Self-Compassion Scale-Short form (SCS[79, 80] will be administered to measure self-compassion. It contains twelve items. It has been shown to have adequate internal consistency, good test re-test reliability and to correlate highly with the longer version of the scale [80].

##### Objective 2.4: Acceptability, Safety and Adherence

Acceptability

Acceptability will be measured using a feedback questionnaire (including quantitative and written responses) at 8-week assessment by a blinded assessor. Participants will be asked to complete the questionnaire and then place it in an envelope and seal the envelope. The feedback questionnaire was developed for the feasibility RCT [39] and adapted for this trial. It consists of written responses asking about what participants thought of the interventions, questions that use a 5-point Likert scale and questions probing contamination. 

Safety

- Current and historic risk to self (suicide and self-harm) and to/from others is evaluated in a semi-structured clinical interview at each assessment time-point. This clinical interview was developed in the feasibility RCT and includes risk to self, from others and to others. Adverse events will be recorded (please see description below).
- There will also be a risk-monitoring question at each intervention session (a single item from the Patient Health Questionnaire-9 items, PHQ-9: item 9). Any change in level of risk during intervention sessions will prompt a risk assessment.

Adherence, Fidelity and Contamination

- The range and average number of sessions completed, total contact time and homework adherence will indicate participant compliance.
- Fidelity and adherence by the therapist will be monitored through clinical supervision. This will include bringing audio recordings of the sessions to supervision. An independent clinical psychologist will rate a random sample (16 tapes, consistent with previous trials, e.g. [81]) against a modified version of the cognitive therapy scale [54] for fidelity, competence, and treatment differentiation. This random sample will be specified by a member of the team not involved in clinical supervision. There are 3 sub-scales to the adherence and competency scale: Scale A consists of non-specific therapy factors (present in both interventions); Scale B is on IMAGINE-specific components and Scale C on NDS-specific components. Scales B and C will be used to assess intervention differentiation. The competency rating ranges from zero (poor) to six (excellent) with a score of three being satisfactory. This evaluation will also indicate whether there had been contamination between the conditions from the therapist having knowledge of both interventions.
- The feedback questionnaire will ask questions probing peer-to-peer contamination, the therapeutic relationship and about therapist motivation.
- The Credibility/Expectancy Questionnaire [55] is a 6-item self-report questionnaire that will be administered to gauge how credible participants believe the intervention to be and whether expectations are matched across the groups. It will be administered following the first session of the intervention, consistent with previous work (e.g. [81]).

### Other measures

#### Baseline measures to characterise the groups

The General Information Questionnaire (GIQ) will be administered at baseline assessment to gather demographic information to characterise the sample. This includes age, postcode (to generate index of multiple deprivation), ethnicity, sex, gender identity, sexual orientation, religion, accommodation and family home composition. This measure was developed in the feasibility RCT [39] and adapted for this RCT. 

#### Screening measures

The Mood and Feelings Questionnaire (long version of the MFQ [56]) will be used to screen for symptoms of depression. This will be a twenty-nine-item version as the four risk items are removed for considerations around mass testing. In addition, there are some optional questionnaires administered during the screen, and these will not be reported in the primary publication. These additional optional pre-randomisation questionnaires are a shorter version of the general information questionnaire, AMI-Y, PIT and the Disturbing Dream and Nightmare Severity Index (DDNSI). The primary aim of these optional measures is to develop the AMI-Y which is a new measure of mental imagery in adolescence. Descriptions of these questionnaires have been reported above except for the Disturbing Dream and Nightmare Severity Index (DDNSI [82]). The DDNSI is a 5-item self-report scale assessing nightmare severity. It has been shown to have good psychometric properties [83]. 

#### Measures of quality of life and service use

Health-related quality of life will be self-reported using the five-dimension version of the EuroQol (EQ-5D-5L; [55]) and the ten-dimension version of the Recovering Quality of Life for users of mental health services (ReQoL-10; [84]). The EQ-5D-5L dimensions cover mobility, self-care, usual activities, pain/discomfort and anxiety/depression and each dimension has 5 levels (no problems, slight problems, moderate problems, severe problems and extreme problems). The ReQoL dimensions cover everyday tasks, trust, coping, doing what I want, happiness, life worth living, enjoyment, hope, loneliness and confidence and each dimension has 5 levels (never, occasionally, sometimes, often, most of the time). Both measures are suitable for adolescents aged 16 and older, with the adult version of the EQ-5D being recommended in preference to the youth version (the EQ-5D-Y) for this age group.

Service use, taking the NHS and social care perspective, will be measured using a brief version of the Child and Adolescent Service Use Schedule (CA-SUS), adapted for the current study and based on previous versions designed for similar populations in schools [85, 86]. The INDIGO CA-SUS will be piloted at baseline and iteratively adapted for use in subsequent follow-up assessments (e.g. if items are not understood, missing or not relevant).

#### Measures in intervention sessions

For clinical purposes, the 12-item version of the MFQ (sMFQ [57]) will be administered at the beginning of each intervention session as well as one item from the PHQ-9 [87] to monitor risk. The sMFQ shows good reliability and content, convergent and concurrent validity [57].

#### Process evaluation

All therapists who deliver the interventions will be invited to take part in the semi-structured interviews. A subset of trial participants will also be invited to take part in semi-structured interviews (estimated sample size of 20 participants) following completion of their participation in the trial. These interviews will be conducted, analysed and reported separately from the main trial.

### Sample size {14}

The primary outcome measure is a continuous measure of depressive symptoms routinely used in NHS settings (MFQ). There is no agreed minimum clinically important difference (MCID) for the MFQ, with a range of five (*d* = 0.47) to ten (*d* = 0.9) points suggested by lived experience representatives (*d* calculated using standard deviations from the IMPACT RCT; Goodyer et al., 2017). Previous research has also suggested that a decrease of approximately ten points on the MFQ is clinically meaningful [44]. To detect the (smaller) effect of *d* = 0.47, with a type-1 error = 0.05 (two tailed), and 80% power, 146 participants are required to be analysed. After inflated for a 10% loss to follow-up (estimate based on the feasibility RCT [39]), 160 will be randomised.

### Recruitment {15}

Strategies identified in our feasibility RCT [39] and co-developed with adolescents and adults with lived experience will be employed to recruit schools and participants within the schools. These strategies aim to improve access to psychological intervention for adolescents with depression and limit teacher burden as much as possible. For example, we will (1) deliver the interventions in the participant’s schools; (2) take an active approach to identifying adolescents with symptoms of depression by offering screening to all of those aged 16 to 18 in a school in stage 1; (3) ask participants about the best way to contact them to discuss the project and directly communicate with the participant following stage 1 to reduce teacher burden and to reinforce our position as external practitioners; (4) not complete formal diagnostic assessments. Furthermore, specific strategies will be discussed with individual schools as to how best to recruit participants and retain them in the trial.

## Methods: assignment of interventions

### Allocation: sequence generation {16a}, concealment mechanism {16b} and implementation {16c}

Following the baseline visit, eligible participants will be randomised using an online King’s Clinical Trials Unit (KCTU) randomisation system. The sequence will be generated using varying permuted block randomisation via a web interface (stratification will be by school) (1:1). The block sizes will not be disclosed to ensure concealment. The chief investigator (VP) will be responsible for logging into the KCTU randomisation system, generating the randomisation and informing the therapist. Randomisation will take place in the period between the baseline assessment and the first intervention session. This period will usually consist of 1 week. The therapist will then deliver the appropriate intervention to the young person.

### Blinding {17a, 17b}

Each follow-up assessment will be carried out by an assessor who is blind to group allocation (and is not the allocated therapist and does not have access to the randomisation system). The senior statistician and all other members of the TMG are blinded to group allocation. The team members who are unblinded to group allocation will be kept at a minimum and will include the chief investigator (VP) and the clinical supervisors (VP and LDR). It is not possible to blind participants due to the nature of the intervention under investigation, and the therapists will be aware of which intervention group participants are allocated to. However, as both experimental and control interventions are credible therapeutic interventions, this should help reduce any potential bias associated with participant expectations of the benefits of the intervention. The two interventions will be referred to as intervention 1 and intervention 2 and both will be described as “programmes aiming to improve low mood and self-esteem” in all participant and staff literature in order to promote equal intervention and credibility between the conditions. That is, participants are not informed as to what is the “new” intervention in order to avoid potential imbalances in expectancy. The primary clinical outcome measure (self-reported depression symptoms) is also less vulnerable to assessor-bias compared to other measures, such as clinician-rated symptom measures. Participants will also be asked not to disclose which intervention they were allocated to during the follow-up assessments. This procedure was followed for the feasibility RCT [39], and no unblinding was recorded. All reasonable attempts will be made to keep school staff blind as to which condition participants have been randomised to. Any unblinding that occurs will be noted by the trial team in an unblinding log and any possible steps taken to reduce unblinding in the future. Given that the chief investigator is unblinded, it is unlikely that emergency unblinding of the assessors will be necessary.

## Methods: retention, data management and analysis

### Participant retention {18b}

Participants will be made aware via the information sheet that their involvement is optional, and they are free to discontinue or withdraw from the intervention and/or RCT at any point without explanation. Adolescents will be asked to call, email or write to the project team should they wish to withdraw from the intervention.

To maximise retention, we will be as flexible as possible with the intervention and study schedule and proactive in resolving any conflicts with the recruited participants or schools/colleges. We will send participants reminders of their sessions and assessments by text message and problem-solve any difficulties attending sessions with them. We will also offer reimbursement for their time spent on the assessments (£15 in Amazon or Love2Shop vouchers per assessment). We will be following the individual school protocols for managing risk and safeguarding and be proactive in forming collaborative relationships with the schools/colleges. For example, we will offer school/college presentations or workshops on mental health (or Psychology).

Reasons for discontinuation or withdrawal will be recorded as thoroughly as possible. Participants who wish to discontinue/withdraw from the intervention, or who are withdrawn by the investigator, will be asked to confirm whether they are still willing to complete the assessments. We will differentiate between those participants who (i) discontinue intervention (but are willing to continue with assessments) or (ii) discontinue the study (including intervention and assessments).

### Data management {19} and confidentiality {27}

The trial database will be managed and maintained by the chief investigator using REDCap. The chief investigator will act as custodian for the trial data. The following guidelines will be adhered to:

- Participant data will be pseudonymised. Participants will be identified on the study database using a unique code and initials.
- All pseudonymised data will be stored on REDCap or on the OneDrive and backed up regularly on the university network.
- All trial data will be handled, computerised and stored in line with the UK Data Protection Act 2018 and UK General Data Protection Regulations (UK GDPR).
- All trial data will be archived in line with Sponsor requirements.
- The quality of the data will be checked using range checks.
- 10% of key eligibility (MFQ score at baseline) and primary outcome data (MFQ score at 8 weeks following randomisation) will be checked against the raw data by someone blind to participant allocation, with further checks if necessary.

### Statistical methods {20a, 20b}

A statistical analysis plan (SAP) has been approved by a blinded senior statistician (BC) and the TSC independent statistician and can be found in supplementary materials. We will report data in line with the Consolidated Standards of Reporting Trials (CONSORT) 2018 Statement for Social and Psychological Interventions. Flow through the trial will also be presented in a standard CONSORT diagram, including number screened, number of adolescents invited to assessment, the numbers completing baseline assessment, numbers of adolescents not eligible and number randomised. Then, by intervention group, the number of participants completing the interventions, the number not beginning the intervention or discontinuing the intervention, the numbers at each assessment time point and the numbers analysed (see Fig. 1).

#### Primary outcome analysis

The primary analysis will be conducted using a mixed-effect multilevel linear regression, fitting a random intercept for: therapist and person. Fixed effects will include as follows: intervention allocated (IMAGINE vs. NDS); baseline severity (MFQ score); school; time by intervention group interaction. This will allow simultaneous modelling of the repeated outcome time points (8 weeks, 16 weeks, 24 weeks), as well as using maximum likelihood estimation to ensure valid inferences in the presence of missing outcome data (under the assumption that data is missing at random). The primary time-point will be at week 8. We will estimate the adjusted mean difference (aMD) with post-estimation commands used to extract treatment group effects at each time-point, for which standardised effect sizes (with 95% confidence intervals) will be reported.

#### Secondary outcomes

Secondary continuous clinical outcomes will be analysed using an approach consistent with the primary outcome analysis. Secondary binary clinical outcomes will be analysed in a manner consistent with the primary outcome but using a logistic regression.

### Analysis population {20c}

In the first instance, analyses will be carried out using the intention to treat principle. This means randomised participants who have provided any post baseline data are included in the analysis and analysed in the groups to which they were randomised, regardless of which intervention they received post-randomisation. Every effort will be made to follow up all participants in both groups for research assessments.

In addition, the Per Protocol Population (PPP) will be assessed. The PPP will analyse the primary outcome using the same analysis described above and will exclude participants who have not adhered to the intervention, i.e. participants who have not completed 3 or more intervention sessions to protocol.

### Missing data {20c}

#### Missing items in scales and sub-scales

Where available we will use missing value guidance provided for scales. Where this is not available, we will prorate missing items only when there are no more than 20% missing items (i.e. for a ten-item questionnaire, prorate only where one or two items are missing) by replacing the missing item values with the mean value of the complete items for each individual. The average value for the complete items will be calculated for that individual and used to replace the missing values. The scale score will be calculated based on the complete values and these replacements. If there are more than 20% missing items, the scale score will be considered missing.

#### Missing baseline data

All efforts will be made to avoid missing baseline data. However, we will summarise and report the number of participants with incomplete baseline measures.

#### Missing outcome data

Missing outcome data will be dealt with by using maximum likelihood methods to fit the mixed models, with participants who only provide baseline measurements being excluded from the analysis. Maximum likelihood estimation provides valid inferences in the presence of missing observations under the assumption that all variables predicting missing outcome data are included in the models and that then the missing data mechanism is ignorable (Missing at Random, MAR).

### Sensitivity analyses

A sensitivity analysis will be carried out on the primary outcome to assess the effect of treatment when assessment timepoints fall outside of the 4-week visit window. Analysis will also be conducted using the Per Protocol Population (PPP). These are further defined in the SAP (please see supplementary material).

### Exploratory outcomes

These will be reported outside of the primary results paper. Significant findings will be interpreted cautiously, acknowledging the issue of multiple testing, need for replication and low statistical power. Please see supplementary material for further detail.

### Health economics

The focus of the economic component of the study is on testing the comprehensiveness, feasibility and acceptability of the measures for application to a future definitive RCT. Statistical tests for differences will not be carried out as this analysis is exploratory. For service use and the two measures of health-related quality of life (EQ-5D-5L and ReQoL-10), we will report descriptive data on the (1) numbers agreeing to complete the measures, (2) data completeness, (3) level of explanation required and (4) any verbal feedback from participants. For service use, this information will be used to make any final amendments to the measure. For health-related quality of life, this information will be used to compare the two measures, including via stated participant preferences between the two measures, which will be actively sought. In addition, service use will be summarised by group as mean, SD and percentage using each item, EQ-5D-5L and ReQoL items will be summarised by group as mean and SD, and quality-adjusted life years (QALYs) will be estimated from the EQ-5D-5L (quality weights not currently available for the ReQoL) and summarised by group as mean and SD.

## Oversight and monitoring

### Trial steering committee and trial management group {5d and 21a}

The Trial Steering Committee (TSC) has been formed and consists of an Independent Chair, two independent psychologists, two experts by experience, an independent statistician, the trial statistician and the chief investigator. The TSC will meet every 6 months (if this is not feasible, the TSC will meet annually as a minimum). Given the scope and nature of the trial, a Data Monitoring Committee {21a} will not be initially formed but the TSC will have the option to initiate the formation of a DMC, if they feel unblinded data needs to be reviewed for the trial to proceed.

The Trial Management Group includes experts in the field (including experts in working with schools, brief psychological interventions, depression, mental imagery) and experts in the methodology and statistics. The TMG will meet every 6 months (if this is not feasible, the TMG will meet annually as a minimum). The chief investigator and her team are responsible for the day-to-day running of the INDIGO trial.

In terms of auditing trial conduct {23}, the chief investigator, along with the other members of the TSC, will monitor the trial to ensure compliance with all applicable regulatory standards and good practice guidelines.

### Plans for communicating important protocol amendments to relevant parties ({25}

Important protocol modifications (e.g. changes to eligibility criteria, key outcome measures) will be discussed with the research team and for major revisions the advice of the Independent Chair of the TSC will be sought. Modifications will also be presented to the wider TSC. Where necessary, Research Ethics Committee (REC) approval will be sought for modifications.

### Stopping guidelines {21b}

The trial may be prematurely discontinued by the Sponsor or chief investigator based on new safety information or for other reasons given by the Ethics Committee, Trial Steering Committee or other regulatory authorities concerned. All adverse events and serious adverse events will be reported to the TSC who will review them. A DMC will not be initially formed but the TSC will have the option to initiate the formation of a DMC if they feel unblinded data needs to be reviewed for the trial to proceed. The TSC will pause the trial to review further if they felt there was a high risk of harmful effects.

The trial may also be prematurely discontinued due to lack of recruitment or upon advice from the Trial Steering Committee, who will advise on whether to continue or discontinue the study and make a recommendation to the sponsor. If the study is prematurely discontinued, active participants will be informed. In consultation with the Independent Chair, next steps will be agreed upon. It is most likely that data collection would continue but that participants would no longer receive the intervention.

### Adverse event reporting and harms {22}

#### Monitoring of adverse events

Potentially anticipated harms and unanticipated harms will be collected systematically from the participants during the clinical interview which includes a risk assessment (e.g. risk to self, to others and from others). This will include, for example asking about suicidal behaviour, self-harm and safeguarding issues. Anticipated harms will be actively assessed at each assessment time point. The clinical interview is also designed to facilitate spontaneous reporting of unanticipated harms, for example by asking about contact with health services which will be followed up by the assessor. If there are harms disclosed in a programme session, this will also be recorded in the clinical interview. There will also be a risk-monitoring question at each intervention session. Any change in level of risk or any indication from the participant of an adverse event will prompt a risk assessment and risk protocols. Contact with services will also be assessed and recorded.

### Reporting responsibilities

Adverse events (AE) will be monitored and recorded in an adverse events form from randomisation to final follow-up (24-week follow-up). These will be reported in the trial publications. The number of serious adverse events and adverse events will be presented as the number of events and number of individuals with events. These will be provided separately for each randomised group. A standard method of reporting will be employed, categorising events by intensity (three levels, mild, moderate and severe). Investigators will also rate whether an event is temporally related to the intervention, rating it in six categories (definitely related, likely to be related, possibly related, unlikely to be related, unrelated, not able to tell).

Any adverse events that are considered by the chief investigator, in consultation with TMG, to be related to trial procedures in any way, or are unexpected, will be reported in a timely fashion to the ethics committee and sponsor. All serious adverse reactions (SAR) or unexpected serious adverse reactions (USAR) will be reported immediately by the chief investigator to the ethics committee and sponsor within 15 days of the chief investigator becoming aware of the event.

#### Pre-defined list of possible adverse events:

The trial will be conducted within a school setting with participants who are reporting symptoms of depression. Possible adverse events (AE) that may be likely in this population:

- Low risk acts of self-harm (not requiring medical attention), e.g. scratching. Possible adverse events (AE) and serious adverse events (SAE) that are less likely but may still occur are:
- High risk acts of self-harm (requiring medical attention, but not medical hospital admission, e.g. deep cuts of the body).
- Admission to a psychiatric hospital.
- Significant and sustained deterioration of a pre-existing mental health condition that required immediate intervention and cannot be accommodated within the treatment protocol (as determined by clinical supervision).
- Sectioned under the Mental Health Act.
- Suicidal behaviour.
- Serious safeguarding issues.
- Death by suicide.

## Discussion

Here we describe a Phase IIb RCT for a brief early intervention for adolescent depression. IMAGINE draws on cognitive science, aiming to build positive future mental imagery, process distressing past negative imagery and improve specificity of autobiographical memories. These processes have been implicated in the development and maintenance of depression but have not previously been combined in a treatment package. Most current gold standard psychological interventions for adolescent depression have been developed for adults and then translated down to younger populations. A strength of the intervention is that it has been co-designed specifically for this age group, with adolescents and adults with lived experience, therapists, parents and teachers. IMAGINE has been designed to be delivered by graduate psychological therapists to hopefully speed future implementation, and to be delivered in schools to improve access.

A strength of the RCT design is in the inclusion of an active control intervention: NDS. This choice of control intervention is to control for factors related to non-specific aspects of therapy, it is based on recommendations in NICE guidelines and ethical considerations of offering an appropriate intervention to adolescents with symptoms of depression. Therefore, all participants are receiving an intervention that aims to reduce symptoms of depression.

If IMAGINE demonstrates efficacy, a future pragmatic effectiveness-implementation hybrid RCT will be indicated. IMAGINE offers the potential for a new intervention that could reduce the associated negative consequences of depression for the individual, their families and wider society. It could expand the repertoire of available effective interventions for depression in adolescence, which has been identified as important to young people and psychological therapists [12].

## Supplementary Information

Supplementary Material 1.

Supplementary Material 2.

## Data Availability

It is intended that the anonymised dataset will be made publicly available following the completion of the trial.

## Abbreviations

IMAGINE: Integrating Memories and Generating Images of New Experiences
INDIGO: INterventions for Depression In younG peOple
CBT: Cognitive behavioural therapy
NDS: Non-directive support
RCT: Randomised controlled trial
OGM: Overgeneral memory
MFQ: Mood and Feelings Questionnaire
KCTU: King’s Clinical Trials Unit
VP: Victoria Pile
NICE: National Institute of Clinical Excellence
SPIRIT: Standard Protocol Items: Recommendations for Interventional Trials
QALYs: Quality adjusted life years
NHS: National Health Service
PPI: Patient and Public Involvement
PTSD: Post-traumatic stress disorder
UK GDPR: UK General Data Protection Regulations
RCADs: Revised Children’s Anxiety and Depression scale
SPPC: Self-worth subscale of the Harter Self-Perception Scale
ISI: Insomnia Severity Index
BADS: Behavioural Activation for Depression Scale
CRIES: Revised Impact of Event Scale: child version
AMI-Y: Assessment of Mental Imagery in Youth
PIT: Prospective Imagery Task
AMT: Autobiographical Memory Task
SCS: Self-Compassion Scale- Short form
PHQ-9: Patient Health Questionnaire-9 items
GIQ: General Information Questionnaire
DDNSI: Disturbing Dream and Nightmare Severity Index
CA-SUS: Child and Adolescent Service Use Schedule
REQoL: Recovering Quality of Life
SAE: Serious adverse events
SAR: Serious adverse reactions
SUSAR: Suspected unexpected serious adverse reaction
AE: Adverse events
AR: Adverse reactions
TSC: Trial steering committee
TMG: Trial Management Group
DMC: Data Management Committee
UK: United Kingdom
CI: Confidence interval
aMD: Adjusted mean difference
SAP: Statistical Analysis Plan
