# Supplementary material for: Evaluating a brief imagery-based intervention for adolescent depression: study protocol for a Phase IIB randomised control trial (INDIGO) in secondary schools
Source: Trials. 2025 Jul 3;26:236. doi: 10.1186/s13063-025-08920-9 (PMC12225131; doi:10.1186/s13063-025-08920-9)
Supplement: Supplementary file 2 — Supplementary Material 2. [file 13063_2025_8920_MOESM2_ESM.pdf]

HARNESSING MENTAL IMAGERY IN A BRIEF SCHOOL-BASED INTERVENTION FOR ADOLESCENT DEPRESSION: A PHASE IIb RANDOMISED CONTROLLED TRIAL (INDIGO)

Statistical analysis plan version 1.0

Trial registration: ISRCTN14015295, registration date: 11.09.23

This SAP has been written based on Protocol Version 1 (11.09.23)

Principal investigator: Victoria Pile

Trial statisticians: Rose Tinch Taylor and Ben Carter

Independent statistician: Peter Martin

Version	Date	Changes
1.0	15.12.2023	First draft of SAP

Victoria Pile<br>Principal investigator	Rose Tinch Taylor<br>Trial statistician	Peter Martin<br>Independent statistician

Contents

ABBREVIATIONS 4

DESCRIPTION OF TRIAL 6

1.1 Principal research objectives 6

1.2 Trial design 6

1.3 Blinding and randomisation procedure 7

1.4 Sample size and power calculations 7

1.5 Eligibility 7

1.6 Trial Interventions 8

MEASURES 8

2.1 General description 8

2.2 Baseline measures 10

2.3 Primary outcome 11

2.4 Secondary outcome measures 11

2.5 Other measures 12

2.6 Adverse Events: 13

2.7 Baseline measures to characterise the groups. 14

DATA ANALYSIS PLAN – DATA DESCRIPTION 16

3.1 Recruitment and representativeness of recruited participants 16

3.2 Baseline comparability of randomised groups 18

3.3 Anticipated intercurrent events 18

3.4 Withdrawal from the intervention and trial 18

3.5 Loss to follow-up and other missing data 18

3.6 Adverse event reporting 18

3.7 Descriptive statistics for outcome measures 18

3.8 Estimation of planning parameters 19

3.9 Adherence to allocated intervention and intervention fidelity 19

3.10 Additional descriptive analysis on Quality of Life and Service Use (reported outside of primary paper) 19

DATA ANALYSIS PLAN – INFERENTIAL ANALYSIS 19

4.1 Definition of primary estimand 20

4.2 Statistical Methods for inferential analysis 20

4.3 Statistical considerations 21

4.4. Quality Assurance 22
4.5 Oversight procedures 23
SOFTWARE 23
5.1. Data management 23
5.2 Statistical software 23
5.3 Calculator for Index Multiple Deprivation 23
5.4 Database and data entry checks 23
APPENDIX 24
Appendix 1: Schedule of Assessments and Measures 24
Appendix 2: Scoring 25
REFERENCES 27

ABBREVIATIONS

A list of abbreviations used throughout the Statistical Analysis Plan

Glossary of terms

AE	Adverse event
AMI-Y	Assessment of Mental Imagery in Youth
AMT	Autobiographical Memory Task
AR	Adverse reaction
BA	Behavioural Activation
BADS	Behavioural Activation for Depression Scale
CAMHS	Child and Adolescent Mental health Services
CA-SUS	Child and Adolescent Service Use Schedule
CI	Chief Investigator
CRIES-8	Child Revised Impact of Event Scale (8 item version)
CONSORT	Consolidated Standards of Reporting Trials
DDNSI	Disturbing Dream and Nightmare Severity Index
EQ-5D-5L	EuroQol-5 Dimensions- 5 Levels
GCP	Good Clinical Practice
GIQ	General Information Questionnaire
ICEs	Intercurrent events
IMAGINE	Integrating Memories and Generating New Experiences (experimental intervention)
INDIGO	INterventions for Depression In younG peOple
ISI	Insomnia Severity Index
KCTU	King's Clinical Trials Unit
lMFQ	Long Mood and Feelings Questionnaire
MAR	Missing at Random

MFQ	Mood and Feelings Questionnaire
NDS	Non-Directive Support (active control intervention)
NHS	National Health Service
NIHR	National Institute for Health and Care Research
PIT	The Prospective Imagery Task
PPP	Per Protocol Analysis
PTSD	Post-traumatic stress disorder
ReQol	Recovering Quality of Life
RCADS	Revised Children's Anxiety and Depression scale
RCT	Randomised Controlled Trial
REC	Research Ethics Committee
SAE	Serious Adverse Event
SAR	Serious Adverse Reaction
sMFQ	Short Mood and Feelings Questionnaire
SCS	Self-Compassion Scale- Short form
SPPC	Self-worth subscale of the Harter Self-Perception Scale
SUSAR	Suspected Unexpected Serious Adverse Reactions
TMF	Trial Master File
TSC	Trial Steering Committee
YP	Young People

STATISTICAL ANALYSIS PLAN (SAP)

DESCRIPTION OF TRIAL

This document details the presentation and analysis strategy for the primary papers reporting results from the INDIGO trial. It is intended that the results reported in these papers will follow the strategy set out herein. Reference is made to the trial protocol version 1 (dated 11.09.23). The SAP has been drafted following the principles of ST:02 Statistical analysis plan part of the KCTU Standard operating procedures.

This statistical analysis plan has been agreed with the Trial Steering Committee Independent Statistician.

1.1 Principal research objectives

1.1.1 Primary objectives

To evaluate whether, in young people aged 16-18 (P), a brief imagery-based intervention (I: IMAGINE; 4 face-to-face sessions) reduces symptoms of depression (O) relative to non-directive support (C: NDS; 4 face-to-face sessions) at 8 weeks following randomisation (T).

1.1.2 Secondary objectives

1. Establish whether any observed changes in symptoms of depression are maintained at 16- and 24-week follow-up.
2. Establish the efficacy of IMAGINE (compared to an active control) on secondary clinical outcomes. This includes changes in anxiety, self-worth, sleep difficulties, school and social impairment, and symptoms of intrusions and avoidance to a negative event.
3. Establish whether there are changes in the key cognitive mechanisms (e.g., emotional mental imagery and memory specificity) and associated mechanisms (self-compassion, activity levels, rumination) when receiving IMAGINE, relative to NDS.
4. To further assess (a) acceptability of the interventions to participants; (b) safety of the interventions; (c) adherence to the intervention protocol by clinicians and participants, fidelity of the intervention and contamination (both therapist and peer to peer contamination).

1.1.3: Exploratory objective

To provide the groundwork for the health economics component of a future pragmatic effectiveness-implementation hybrid RCT (embedded into NHS settings). This includes assessing the comprehensiveness, feasibility, and acceptability of collecting economic measures of service use (for the estimation of cost per participant) and health-related quality of life (for the calculation of quality adjusted life years; QALYs).

1.2 Trial design

INDIGO is a phase IIb randomised controlled trial (RCT; n=160) taking place in secondary schools in the UK. INDIGO is a multi-school, assessor-only blinded, parallel group superiority RCT comparing IMAGINE versus active control (non-directive support; NDS). Participants will be randomly allocated to IMAGINE or NDS (control) in a 1:1 allocation ratio in a population of young people in schools presenting with symptoms of depression, to determine superiority of IMAGINE versus NDS at eight weeks post randomisation.

1.3 Blinding and randomisation procedure

Each assessment will be carried out by an assessor who is blind to intervention allocation. It is not possible to blind participants due to the nature of the intervention under investigation (the trial therapists will be aware of which intervention group participants are allocated to). However, as both experimental and control interventions are credible therapeutic interventions this should help reduce any potential bias associated with patient expectations of the benefits of the intervention. All reasonable attempts will be made to keep school staff blind as to which condition participants have been allocated to. In their final sessions of IMAGINE or NDS, blinding will be explained to the participants by the therapist. The therapist will ask participants not to disclose which intervention they were allocated to during the follow up assessments. This procedure was followed for the feasibility RCT and no unblinding was recorded. Planned and unplanned unblinding events will be recorded in a unblinding log. CI (VP) who will be conducting the analysis was fully blind to outcome data until the SAP was signed off. In line with KCTU statistical SOP on blinding ST-06 V3.1, the statistician/analyst may be unblinded once the SAP has been signed off.

Once baseline assessments are complete, the individuals will be randomised to one of the intervention groups. Randomisation will be done in a 1:1 ratio. Randomisation is at the individual level. Eligible participants will be randomised using a masked web based 'KCTU randomisation and IMP management system', maintained by the King's Clinical Trials Unit (KCTU) for the duration of the project.

The sequence will be generated using varying permuted block randomisation via a web interface (stratification will be by school). The block sizes will not be disclosed to ensure concealment. The procedure is as follows: the chief investigator (VP) will be responsible for logging into the KCTU randomisation system, generating the randomisation and informing the therapist. VP will electronically submit details of each participant to the KCTU system. This includes participant ID number, school, initials and age at consent. The system immediately notifies VP and records the randomisation outcome. This system can only be accessed by trial staff who are trained and have previously been allocated a username and password. Requests for passwords are via the trial manager to the KCTU.

1.4 Sample size and power calculations

The sample size is n=160 based on the following considerations. We have assumed ICC = 0, a conservative approach for power analysis. The primary outcome measure is a continuous measure of depressive symptoms routinely used in NHS settings (MFQ). There is no agreed minimum clinically important difference (MCID) for the MFQ, with a range of five (d=0.47) to ten (d=0.9) points suggested by the literature and lived experience representatives (d calculated using standard deviations from the IMPACT RCT (Goodyer et al, 2017)). To detect this effect (d=0.47), with a type-1 error=0.05 (two tailed), and 80% power, 144 are required to be analysed. After inflating for a 10% loss to follow up 160 will be randomised.

1.5 Eligibility

<u>1.5.1 Inclusion</u>

(1) Aged 16-18;

(2) Able to provide Informed consent;

(3) Willing and able to engage in psychological therapy and complete assessments;

(4) Scoring above clinical cut-off on Mood and Feelings Questionnaire at both screen and baseline assessment (MFQ of 29 items at screen, clinical cut-off ≥17; MFQ of 33 items at baseline, clinical cut-off ≥20).

<u>1.5.2 Exclusion</u>

(1) Diagnosis of learning disability or significant head injury, neurological disorder or epilepsy;

(2) Unable to fluently communicate in spoken English;

(3) Currently receiving another psychological intervention (including school counselling);

(4) Moderate to high levels of risk. This will be verbally assessed with the participant at first interview and discussed in supervision with the Chief Investigator (VP). This will be based on clinical judgement, but an outline is that Imagery Rescripting is unlikely to be appropriate for young people presenting with current and/or significant self-harm (e.g., which requires medical attention); active suicidal ideation and an active plan to harm themselves and; those presenting with significant risk to others. The clinical decision will be informed by their history of risk.

(5) Current diagnosis of bipolar disorder, PTSD or psychosis. This will be stated in the information sheet and verbally assessed with the participant at first interview.

(6) Other significant conditions or factors that contraindicate the individual's participation in the trial.

1.6 Trial Interventions

Both interventions will consist of three to four individual sessions. Sessions will last up to a maximum of 90 minutes, with the possibility of young people taking breaks during the session if needed. A maximum of four 90-minute sessions will be offered.

Experimental intervention, IMAGINE (Integrating Memories And Generating New Experiences): IMAGINE will follow an intervention manual and will be accompanied by a therapy workbook. The intervention will combine (A) imagery protocols to reduce the distress associated with negative images and build positive future images and (B) Memory Specificity Training to increase specificity and access to memories.

Control intervention, Non-Directive Support (NDS): NDS will follow intervention guidelines. NDS is designed to control for factors that, other than active components of therapy, could contribute to change such as passage of time and non-specific aspects of therapy (e.g., speaking to an empathic therapist). NDS is a variant of counselling, designed to be matched for contact time and frequency of sessions and to control for non-specific factors in therapy that contribute to change (e.g. speaking to an empathic therapist).

Listing of concomitant therapies

Concurrent psychological therapy is an exclusion criterion for entry into the trial. However, it is possible that young people begin alternative interventions during the follow-up period. Psychological therapies and medication for mental health will be recorded and reported per intervention group.

MEASURES

This section contains an overview of those measures relevant to the quantitative analysis. Please also refer to the schedule of assessments (Table 1 and Appendix 1) and a brief description of instrument scoring (Appendix 2) for further description of timings of assessments and information on scoring algorithms.

2.1 General description

- Depression will be measured using a self-report measure of depression (MFQ). The MFQ is a well-validated and widely used measure of depression in NHS and research settings. It demonstrated excellent internal consistency, high test re-test reliability as well as concurrent and convergent validity (Thabrew et al., 2018). The child self-report version will be used (Costello, 1988).

- The General Information Questionnaire (GIQ) gathers demographic information to characterise the sample. This includes age, postcode (to generate index of multiple deprivation), ethnicity, sex, gender identity, sexual orientation, religion, accommodation and family home composition. This measure was developed in the feasibility RCT (Pile et al., 2021) and adapted for this RCT.
- The anxiety subscales from the Revised Children's Anxiety and Depression scale (RCADs; Chorpita et al., 2005) will be administered to measure anxiety. This is a youth self-report questionnaire. The anxiety subscales will be administered: separation anxiety disorder, social phobia, generalised anxiety disorder, panic disorder and obsessive-compulsive disorder. This provides a total Anxiety Scale (sum of 5 anxiety subscales) and individual subscale scores. It has been shown to have good internal consistency, test-re-test reliability and concurrent and convergent validity.
- The Self-worth subscale of the Harter Self-Perception Scale (SPPC; Harter S, 1999) will be administered to measure general self-worth. The Harter Self-Perception Scale is a self-report questionnaire with five subscales but only the self-worth subscale will be administered. The self-worth subscale consists of five items. The Harter Self-Perception Scale demonstrates strong psychometric properties (Harter, 2012).
- The Insomnia Severity Index ISI, (Bastien et al., 2001) consists of seven items rating insomnia (0–4 Likert scale), with the summed total (between 0 and 28) indicating moderate (15–21) or severe (22–28) clinical insomnia. There is one version that is used across the age range and has been shown to be valid and reliable in young people (Short et al., 2013)
- The Behavioural Activation for Depression Scale (BADS; Kanter et al., 2007) will measure school and social impairment as well as activation and avoidance/rumination. The BADS is a 25-item questionnaire comprising four subscales: Activation; Avoidance/Rumination; School Impairment; and Social Impairment. Participants rate statements according to how true they were for them during the past week, on a scale from 0 (*Not at all*) to 6 (*Completely*). [The BADS was originally designed to measure the kinds of behaviours hypothesised to be responsible for symptom change during Behavioural Activation treatment for depression (BA).]
- The eight-item version of the Child Revised Impact of Event Scale (CRIES; Perrin et al., 2005) will be administered to measure symptoms of intrusions and avoidance in reference to a negative event. The CRIES has been established as a good screen for PTSD with good internal reliability.
- The Assessment of Mental Imagery in Youth (AMI-Y) is a questionnaire measure co-developed for the trial with young people and based on the process evaluation for IMAGINE (Pile et al., 2023) and the *Imagery Interview* (Hackmann et al., 2000). Following a general description of mental imagery, participants are asked to identify one positive and one negative spontaneous mental image and rate the image on a number of criteria including frequency and controllability. There are therefore two scales, one for a positive image and one for a negative image.
- The Prospective Imagery Task (PIT, based on Stober, 2000; adapted for use in young people, Pile & Lau, 2018) will measure prospective emotional mental imagery. Participants are asked to read fourteen scenarios (e.g., "You will make good and lasting friendships"), imagine each happening to them and then rate their generated mental image on a five-point scale (from 'No image at all' to 'Very clear and detailed'). The scale includes seven negative and seven positive scenarios and there are therefore two subscales (negative imagery and positive imagery).

- Autobiographical Memory Task (AMT, Williams & Broadbent, 1986) will be administered to measure memory specificity. Participants will be provided with an example of a specific memory and asked to give a specific memory to ten cue words (five positive; five negative).
- Self-Compassion Scale- Short form (SCS, Neff, 2003; Raes et al., 2011) is a twelve-item measure of self-compassion. It has been shown to have adequate internal consistency and near perfect correlation with the longer version of the scale.
- Child and Adolescent Service Use Schedule (CA-SUS) will be administered to measure service use. A new version of the CA-SUS will be developed based on versions developed for adolescent depression populations but adapted specifically for this trial. A brief, semi-structured interview measure has been designed. Young people will be asked whether they have made use of each service listed, with follow-up questions to understand the amount of use. (This data will be reported outside of the primary paper).
- The EQ-5D-5L (Herdman et al., 2011) and the Recovering Quality of Life (ReQoL; Keetharuth et al., 2018) will be administered to measure health-related quality of life. The EQ-5D-5L has five dimensions (mobility, self-care, usual activities, pain/discomfort and anxiety/depression) and each dimension has 5 levels (no problems, slight problems, moderate problems, severe problems and extreme problems). The ReQoL consists of 10 items (including "I could do the things I wanted to do"; "I felt confident in myself") each with a 5-level response category. (This data will be reported outside of the primary paper).
- A clinical interview called 'Involvement with mental health services and risk assessment' will assess the participants history of diagnoses, current and past medication, visits to GP, CAMHS involvement, previous psychological therapies and visits to A&E about their mental health. It will also assess (and quantify) levels of current and historic risk to self (suicide and self-harm), to others and from others. The assessor will rate level of risk on a five-point scale (Low; Low/Moderate; Moderate; Moderate/High; High). The definition of these categories was developed in the feasibility RCT. At 8-, 16- and 24-week assessment it will assess any change in risk since baseline assessment.
- The feedback questionnaire was developed for the feasibility RCT (Pile et al., 2021) and adapted for this trial. It consists of written responses asking about what young people thought of the programmes, questions that use a 5-point Likert scale and questions probing contamination. The questions include those that ask about the therapeutic relationship and about therapist motivation.
- The Credibility/Expectancy Questionnaire (Devilly & Borkovec, 2000) is a 6-item self-report questionnaire that measures participant beliefs about the potential effectiveness of the intervention. It will be administered following the first session of therapy, consistent with previous work (e.g., Freeman et al., 2021).

2.2 Baseline measures

Outcomes will be assessed following the schedule of outcome assessments (Table 1). Note, some measures are gathered at the screening visit (see Table 1 and Appendix 1).

- Demographics/Participant characteristics:
    - Age
    - Ethnicity
    - English as first language

    - Sex
    - Gender identity
    - Number of GCSEs and BTechs
    - Free school meals
    - Presence of Education, Health and Care Plan
    - Sexual orientation
    - Index of multiple deprivation (listed in software section)
    - Accommodation
    - Family home composition
    - Religion
- Depression (MFQ, 33-item version)
- Anxiety (RCADS, anxiety subscales)
- Self-worth (SPPC)
- Sleep difficulties (ISI)
- Activity levels, rumination, school and social impairment (BADS – 25 item)
- Distress/post-traumatic stress symptoms to a negative event (CRIES-8)
- Mental Imagery (AMI-Y)
- Future imagery vividness (PIT)
- Memory specificity (AMT)
- Self-Compassion (SCS)
- Measures of Quality of life (EQ-5D-5L; ReQoL), service use (CA-SUS) and acceptability of these measures (including assessor rated level of explanation and participant rated preference)
- Involvement with mental health services (history of diagnoses, current and past medication, visits to GP, CAMHS involvement, previous psychological therapies, and visits to A&E about their mental health) and level of risk.

2.3 Primary outcome

Mood and Feelings Questionnaire (long version, MFQ) at 8 weeks following randomisation.

2.4 Secondary outcome measures

All measures will be collected at four time points: baseline, 8, 16 and 24 weeks after randomisation.

- MFQ at 16 and 24 weeks after randomisation.
- Anxiety (RCADS) at 8, 16 and 24 weeks after randomisation.
- Self-worth (SPPC) at 8, 16 and 24 weeks after randomisation.

- Sleep difficulties (ISI) at 8, 16 and 24 weeks after randomisation.
- Activity levels, rumination, school and social impairment (BADS – 25 item) at 8, 16 and 24 weeks after randomisation.
- Distress/post-traumatic stress symptoms to a negative event (CRIES-8) at 8, 16 and 24 weeks after randomisation.
- Mental Imagery (AMI-Y; positive and negative subscales) at 8, 16 and 24 weeks after randomisation.
- Future imagery vividness (PIT; positive and negative subscales) at 8, 16 and 24 weeks after randomisation.
- Memory specificity (AMT) at 8, 16 and 24 weeks after randomisation.
- Self-Compassion (SCS) at 8, 16 and 24 weeks after randomisation.

2.5 Other measures

2.5.1 Acceptability

Acceptability will be measured using a feedback questionnaire (including quantitative and written responses) at 8-week assessment by a blinded assessor. Participants will be asked to complete the questionnaire and then place in an envelope and seal the envelope.

2.5.2 Safety

- Current and historic risk to self (suicide and self-harm) and to/from others is evaluated in a semi-structured clinical interview at each assessment time-point and quantified with a severity score. At 8-, 16- and 24-week assessment, this constitutes any change in risk since the previous assessment.
- There will also be a risk-monitoring question at each therapy session (a single item from the PHQ-9). Any change in level of risk during therapy sessions will prompt a risk assessment.

2.5.3 Adherence, Fidelity and Contamination

- The range and average number of sessions completed, total contact time and homework adherence.
- Fidelity and adherence by the therapist will be monitored through clinical supervision. This will include bringing audio recordings of the therapy sessions to supervision. An independent clinical psychologist will rate a random sample [16 tapes, consistent with previous trials (e.g. Freeman et al, 2021)] against a modified version of the cognitive therapy scale (Vallis et al., 1986) for fidelity, competence, and treatment differentiation. There are 3 sub-scales to the adherence and competency scale: Scale A consists of non-specific therapy factors (present in both interventions); Scale B is on IMAGINE-specific components and Scale C on NDS-specific components. The competency rating ranges from zero (poor) to six (excellent) with a score of three being satisfactory. This evaluation will also indicate whether there had been contamination between the conditions from the therapist having knowledge of both interventions.
- The feedback questionnaire will ask questions probing peer-to-peer contamination, the therapeutic relationship and about therapist motivation.
- The Credibility/Expectancy Questionnaire (Devilly & Borkovec, 2000) will be administered following the first session of the therapy to gauge how credible young people believe the intervention to be and whether expectations are matched across the groups.

2.5.4 Screening measures

The Mood and Feelings Questionnaire (long version, MFQ, Angold et al., 1995) will be used to screen for symptoms of depression. This will be a twenty -nine item version as the four risk items are removed for considerations around mass testing.

In addition, there are some optional questionnaires administered during the screen and these will not be reported in the primary publication. These additional optional pre-randomisation questionnaires are a shorter version of the general information questionnaire, AMI-Y, PIT and the Disturbing Dream and Nightmare Severity Index (DDNSI). The primary aim of these optional measures is to develop the AMI-Y which is a new measure of mental imagery in young people. Descriptions of these questionnaires have been previously reported except for the Disturbing Dream and Nightmare Severity Index (DDNSI). The DDNSI is a 5-item self-report scale assessing nightmare severity. The total score ranges from 0 to 37, with higher scores indicating a more severe problem. The internal consistency of the scale in a large sample of over 3000 students was very good ($\alpha = 0.91$).

2.5.5 Measures of quality of life and service use

This data will be reported outside of the primary paper.

- The EQ-5D-5L and ReQoL will measure quality of life and service use will be measured using a semi-structured interview, the CA-SUS.
- The relative feasibility and acceptability of these measures will be assessed by: (1) numbers agreeing to complete each measure, (2) data completeness, (3) level of explanation required and (4) verbal feedback on preferences between the two measures (actively sought).

2.6 Adverse Events:

- Adverse events (AE), adverse reactions (AR), serious adverse events (SAE) and serious adverse reactions (SAR) will be summarised. All adverse events will be recorded and reported using an adverse events form.
- Potential adverse events could be reported by participants during therapy and at each assessment time-point (8-weeks, 16-weeks, and 24-weeks post-randomisation). Researchers will record these in the separate researcher-entered adverse events form in the main database.
- The occurrence of adverse events (AEs) will be monitored actively and systematically, following CONSORT guidance for reporting harms. Serious adverse Event (SAE), Serious Adverse Reaction (SAR) or Unexpected Serious Adverse Reaction (USAR) are defined as any adverse event, adverse reaction or unexpected adverse reaction, respectively, that results in death, is life-threatening, required overnight hospital admission or prolongation of existing hospitalisation (this does not include A&E attendance and discharge without admission), results in persistent or significant disability or incapacity or consists of a congenital anomaly or birth defect.
- A standard method of reporting will be employed, categorising events by intensity (three levels, mild, moderate and severe). Investigators will also rate whether an event is temporally related to the intervention, rating it in six categories (definitely related, likely to be related, possibly related, unlikely to be related, unrelated, not able to tell).

2.7 Baseline measures to characterise the groups.

A general information questionnaire will be completed at baseline, The General Information Questionnaire gathers demographic information, including age, postcode (to generate index of multiple deprivation) ethnicity, sex, gender identity, sexual orientation, religion, accommodation and family home composition.

2.8 Measures in therapy sessions

For clinical purposes, the shorter version of the MFQ (sMFQ, 12 items) will be administered at the beginning of each intervention session as well as one item from the PHQ-9 to monitor risk.

Table 1: Schedule of outcome assessments

	STUDY PERIOD					
	Pre-study Screening/ consent	Baseline/ randomisation	Following randomisation			End of Study
			8-weeks ±4 weeks	16-weeks ±4 weeks	24- weeks ±4 weeks	
TIMEPOINT	-1	t_0	t_1	t_2	f_1	
ENROLMENT:						
Eligibility screen	X	X				
Informed consent	X	X				
Demographic/ clinical characteristics		X				
Random Allocation		X				
ASSESSMENTS:						
MFQ (29 items)	X					
MFQ (33 items)		X	X	X	X	
RCADS		X	X	X	X	
BADS		X	X	X	X	
AMI-Y	(X)	X	X	X	X	
SPPC		X	X	X	X	
PIT	(X)	X	X	X	X	
CRIES-8		X	X	X	X	
SCS		X	X	X	X	
ISI		X	X	X	X	
AMT		X	X	X	X	
DDNSI	(X)					
GIQ (short version)	(X)					
GIQ (full version)		X				
EQ-5D-5L and ReQoL		(X)	(X)	(X)	(X)	
CA-SUS		(X)	(X)	(X)	(X)	
Involvement with mental health services and risk assessment		X	X	X	X	
Feedback questionnaire			X			
Adherence and fidelity						X
INTERVENTION:						
IMAGINE		◆———	———◆			
Control: NDS		◆———	———◆			
sMFQ (12 items) and PHQ-9 risk item		◆———	———◆			

(X) not included in primary paper

DATA ANALYSIS PLAN – DATA DESCRIPTION

Analyses will be carried out by the Chief Investigator, Dr Victoria Pile as part of her NIHR Advanced Fellowship (under the supervision of the trial statisticians).

3.1 Recruitment and representativeness of recruited participants

We will report data in line with the Consolidated Standards of Reporting Trials (CONSORT) 2018 Statement for Social and Psychological Interventions. This will include the number of young people screened, number of young people invited to assessment, the numbers completing baseline assessment and numbers of young people not eligible and number randomised. Then, by intervention group, the number of young people completing the programmes, the number not beginning the programme or discontinuing the programme, the numbers at each assessment time point and the numbers analysed (see Figure 1).

Figure 1: Flow through trial in CONSORT diagram

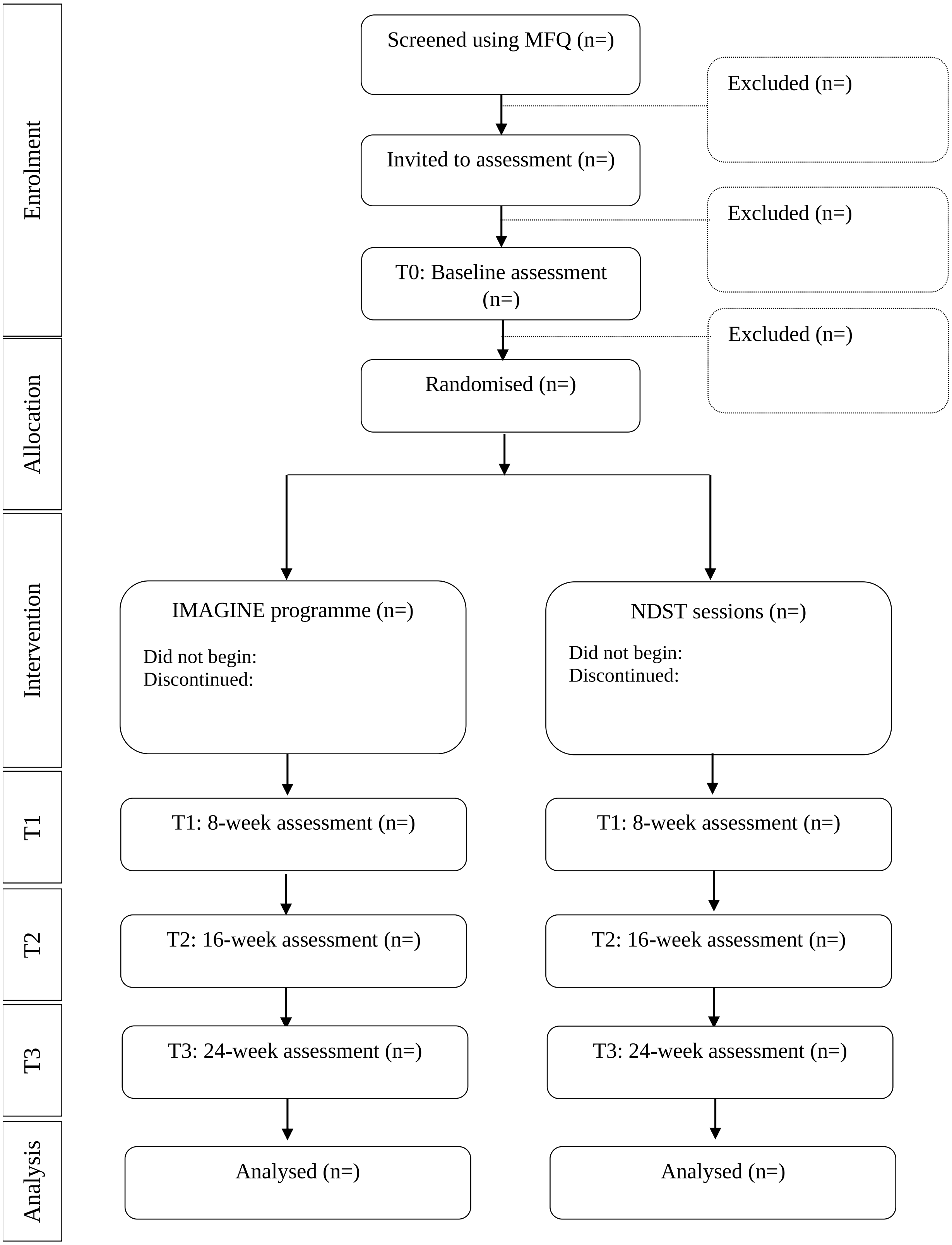

3.2 Baseline comparability of randomised groups

Summary statistics describing participants overall and by intervention group will be presented for baseline values (Section 2.2). These will include counts and percentages for binary and categorical variables, and means and standard deviations, or medians with lower and upper quartiles, for continuous variables, along with minimum and maximum values and counts of missing values. There will be no tests of statistical significance or confidence intervals for differences between randomised groups on any baseline variable. The baseline characteristics of those missing follow up will be compared to those with complete follow up data.

3.3 Anticipated intercurrent events

Intercurrent events (ICEs) are events that occur after randomisation and affect the occurrence or interpretation of outcome measures after the event such as death, expelled from school or accessing CAMHS or other mental health services.

Withdrawal from data collection (e.g. withdrawal of consent or uncontactable) also affects the outcomes as later measurements will be missing. However, as this affects the analysis and not the estimand, it is not considered an intercurrent event.

3.4 Withdrawal from the intervention and trial

We will separately summarise the number and proportion of participants who actively withdraw from the trial and, if possible, their reasons for withdrawal, overall and by group. (In line with our ethical informed consent procedure, participants can discontinue or withdraw from the study at any time without giving a reason).

3.5 Loss to follow-up and other missing data

The number and proportion of participants missing each primary and secondary outcome variable will be presented by intervention group and overall, at each time point.

3.6 Adverse event reporting

The number of serious adverse events and adverse events will be presented as the number of events and number of individuals with events. These will be provided separately for each randomised group. Adverse events will only be presented descriptively. All AEs/SAEs will be summarised and reported in the Open report for the joint Data Monitoring Committee (DMC)/ Trial Steering Committee (TSC). SAEs will also be circulated to the TSC chair for review. Action will then be taken accordingly depending on implications for the conduct of the trial.

3.7 Descriptive statistics for outcome measures

We will summarise primary and secondary outcomes overall and by intervention group at 8-weeks, 16-weeks, and 24-weeks post-randomisation, where available. We will report categorical variables as frequencies and proportions, continuous variables using means and SD, and medians and IQR. We have defined outcome measures above (please refer to Sections 2.3 and 2.4).

In addition, descriptive statistics for the therapy measures (short version of the MFQ, 12 items) and additional risk item from PHQ9) will be reported per session number and per group.

3.8 Estimation of planning parameters

To describe how strongly participants in the same school resemble each other we will calculate the Intracluster Correlation Coefficient (ICC) of the primary outcome measure listed in section 2.3 at 8-week time-point by intervention group.

3.9 Adherence to allocated intervention and intervention fidelity

For a participant in either intervention group to be considered as adhering to the intervention, they must attend 3 or more sessions of the programme. We will report the number and proportion of participants meeting the adherence criterion out of all of those allocated to each group, and out of those who attended at least one session.

Descriptive statistics will be used to summarize assessments of feasibility and acceptability in terms of recruitment, drop-out and completeness of therapy. Outcomes at 8, 16 and 24 weeks will be presented separately for each group and summarised using counts and percentages for binary and categorical variables, and means and standard deviations for continuous variables, along with counts of missing values.

3.10 Additional descriptive analysis on Quality of Life and Service Use (reported outside of primary paper)

Service use, taking the NHS and social care perspective, will be measured using the CA-SUS, adapted for the current study and based on previous versions designed for depression populations. We will further adapt the measure iteratively throughout the course of the trial, based on feedback from participants and descriptively report changes to the measure. Service use data will be summarised by group (mean, SD and percentage using). Statistical tests for differences will not be carried out as this analysis is exploratory. The focus is on testing the comprehensiveness, feasibility and acceptability of the measure for application in a future definitive RCT.

For the comparison of the two effectiveness measures (ReQoL and EQ-5D-5L), we will report descriptive data on the:

(1) Numbers agreeing to complete the measures: The relative percentage of participants agreeing to complete each measure at each timepoint will be reported. 80% of young people agreeing to complete each measure is deemed feasible.
(2) Data completeness: The number of participants that have completed at least 80% of the items on each questionnaire. 80% completeness is deemed feasible for each measure and the relative completeness for each measure will be reported for each timepoint.
(3) Level of explanation required: The assessor will rate each measure for the level of explanation required by the participant on a three-point Likert scale (0, 1, 2). Descriptive data will be reported for each measure at each timepoint.
(4) Verbal feedback on preferences between the two measures (actively sought). Participant preference will be reported descriptively for each assessment timepoint.

In addition, QALY's will be calculated using scores from the EQ-5D and reported descriptively by group as well as scores from the ReQoL.

DATA ANALYSIS PLAN – INFERENTIAL ANALYSIS

In the first instance, analyses will be carried out using the intention to treat principle. This means all randomised participants (eligibility as defined in Section 1.5) are included in the analysis and analysed in the groups to which

they were randomised, regardless of which intervention they received post-randomisation. Every effort will be made to follow up all participants in both groups for research assessments.

As the primary and secondary outcomes are continuous, we will report the mean differences (MD) in instrument scores between the intervention (IMAGINE) and control (NDS) groups and their associated two-sided 95% confidence intervals, adjusting for the baseline of the outcome. These results will be estimated using linear mixed-effects models with all post-randomisation measures as dependent variables. Section 4.2 describes the models we will use for the primary and secondary outcomes, respectively. The significance level will be 5% (two-sided) for the primary outcome as well as for the secondary outcomes.

4.1 Definition of primary estimand

The primary estimand is the baseline adjusted mean difference (aMD) (and associated confidence interval) in participant-reported MFQ, measured at 8 weeks post-randomisation; between IMAGINE, an imagery-based psychological intervention, and the NDS group; in young people (age 16 to 18) presenting with symptoms of depression; with all randomised participants included regardless of the occurrence of any intercurrent events (i.e., treatment policy/intention to treat approach)

The estimand is described by the following attributes:

- Population: Young people aged 16 to 18 in school scoring above the clinical cut off on the MFQ and who meet the trial inclusion/exclusion criteria.
- Endpoint (primary): Depression (measured using the 33-item MFQ) at 8-weeks post-randomisation.
- Interventions: A novel psychological intervention, Integrating Memories and Generating New Experiences (IMAGINE), which targets core cognitive factors implicated in depression (dysfunctional mental imagery and maladaptive memory processes), compared to non-directive support (NDS), regardless of discontinuation for any reason (treatment policy strategy).
- Population-level summary: Adjusted Mean Difference (aMD) on the raw scale with 95% confidence intervals and p-value.

4.2 Statistical Methods for inferential analysis

<u>4.2.1 Primary outcome</u>

The main statistical analyses will estimate the aMD MFQ scores between patients randomised to IMAGINE and NDS by intention to treat at 8-weeks following randomisation. Group difference estimates and associated confidence intervals will be reported. We will conduct the primary analysis after all follow-up data is collected.

<u>Primary outcome analysis</u>

The primary analysis will be conducted using a mixed-effect multilevel linear regression, fitting a random intercept for: therapist and person. Fixed effects will include: intervention allocated (IMAGINE vs. NDS); baseline severity (MFQ score); school; time by intervention group interaction. This will allow simultaneous modelling of the repeated outcome time points (8-weeks, 16-weeks, 24-weeks), as well as using maximum likelihood estimation to ensure valid inferences in the presence of missing outcome data (under the assumption that data is missing at random). The adjusted mean difference (aMD) with post-estimation commands will be used to extract treatment group effects at each time-point, for which standardised effect sizes (with 95% confidence intervals) will be reported.

<u>4.2.2 Secondary Outcomes</u>

Secondary continuous clinical outcomes will be analysed using an approach consistent with the primary outcome analysis. Secondary binary clinical outcomes will be analysed in a manner consistent with the primary outcome but using a logistic regression.

4.3 Statistical considerations

4.3.1 Time points

Outcomes are measured at baseline, 8, 16, and 24-weeks post-randomisation.

4.3.2 Stratification and clustering

The randomisation of individual participants is stratified by school. Therefore, we will incorporate this stratification variable into all models as independent variables. However, if there are a small number of participants within schools, then there may be issues with convergence of model's sparse data. Non-convergence may also occur due to small numbers of participants per therapist, in this case the therapist level may be dropped. Any convergence issues will be resolved by a fully blinded Senior statistician.

The data structure for the continuous outcomes is longitudinal with repeated measures over time. To account for the within-participant correlation, we will perform linear mixed effects models with all repeated measures as dependent variables, with the addition of a random effect at the participant level. We will also add a random effect at the therapist level to account for within-therapist correlation (please refer to Sections 4.2.1 and 4.2.2).

4.3.3 Missing items in scales and sub-scales

Where available we will use missing value guidance provided for scales. Where this is not available, we will prorate missing items only when there are no more than 20% missing items (i.e., for a ten-item questionnaire, prorate only where one or two items are missing) by replacing the missing item values with the mean value of the complete items for each individual. The average value for the complete items will be calculated for that individual and used to replace the missing values. The scale score will be calculated based on the complete values and these replacements. If there are more than 20% missing items, the scale score will be considered missing.

4.3.4 Missing baseline data

All efforts will be made to avoid missing baseline data. However, we will summarise and report the number of participants with incomplete baseline measures.

4.3.5 Missing outcome data

Missing outcome data will be dealt with by using maximum likelihood methods to fit the mixed models described in Sections 4.2.1 and 4.2.2, with participants who only provide baseline measurements being excluded from the analysis. Maximum likelihood estimation provides valid inferences in the presence of missing observations under the assumption that all variables predicting missing outcome data are included in the models and that then the missing data mechanism is ignorable (Missing at Random, MAR).

4.3.6 Method for handling multiple comparisons

We have a single primary outcome at week 8, so no adjustments for multiple comparisons is needed. No adjustment will be made for the secondary outcome assessments. We will make this explicit to the reader in the final publication.

4.3.7 Method for handling non-compliance (per protocol analysis)

The Per Protocol Population (PPP) will be assessed and excludes participants defined as protocol violators (those that fail fidelity). The PPP will analyse the primary outcome using the same analysis described in 3.2.1 above and will exclude participants who have not adhered to the intervention, i.e. participants who have not completed 3 or more sessions of the programme will be excluded from the analysis.

4.3.8 Parametric Assumption Checks

Model assumptions will assess whether residuals are identically, independently, normally distributed with a zero mean and constant variance. Transformations will be considered where data may not be normally distributed.

4.3.9 Sensitivity analyses

A sensitivity analysis will be carried out on the primary outcome to assess the effect of treatment when assessment timepoints fall outside of the +/- 4-week visit window. We will create an indicator variable: 1=within +/- 4-week visit window, 0=not within +/- 4-week window, and then re-run the primary analysis model (for the MFQ) for only those with the indicator variable=1.

4.3.10 Planned exploratory analyses

The study is not formally powered for moderation analyses or subgroup analysis, but we will investigate in an exploratory fashion whether 8-week depression (as measured by the MFQ) intervention effects differ by the below variables:

1. Sex at birth, 2 groups: male; female.
2. Levels of baseline depression, 2 groups based on a higher baseline MFQ cut-off score of 29 (Daviss et al., 2006; Eg et al., 2018): score of 20 to 28; score of 29 or above.
3. Insomnia severity, 2 groups based on an ISI cut-off score of 15 at baseline (Bastien et al., 2001): score of 14 or below; score of 15 and above relating to moderate and severe difficulties.
4. Levels of baseline CRIES score, 2 groups based on a cut-off score of 17 (Perrin et al., 2005): score of 16 and below; score of 17 and above.

4.3.11 Interim analyses

No interim analyses are planned for this study.

4.4. Quality Assurance

KCTU SOPS will be followed where possible.

4.4.1 Quality control

The chief investigator (VP) will carry out data checks on REDCAP data prior to final data lock.

4.4.2 Approval and version control of the SAP

As per KCTU Statistical SOP ST-02 Statistical Analysis Plan, after initial sign off of the SAP, if any changes are required, these will be decided by the blinded Statistician and not by the unblinded CI, so as to avoid any chance or appearance of introducing bias having seen unblinded data. A copy of the tracked changes version will be retained, and the updated version will be approved by the Chief Investigator(s), Statistician and TSC statistician. Any changes between versions will also be summarised on the cover page. The initial signed off version will be versioned as Version 1.0. Minor amendments following this should be versioned with sub-numbering e.g., Version 1.1, Version 1.2 etc. Major amendments should be numbered as Version 2.0, 3.0 etc.

4.5 Oversight procedures

A combined TSC and DMC will be overseeing the combined data throughout the trial. The composition of the TSC/DMC is an Independent Chair, an Independent Trial Statistician, two Lived Experience Representatives and two Independent Psychologists as well as the Chief Investigator and the Trial Statistician.

SOFTWARE

5.1. Data management

REDCap will be used to manage the trial database; this is hosted on a dedicated server at KCL. Data extracts will be obtained by VP in comma separated (.csv) periodically as needed and at the end of the study. The KCTU bespoke randomisation system will be used for randomisation and is also hosted on a dedicated server at KCL and managed by the King's CTU. The KCTU will extract randomisation data periodically as needed by the research team and provide in Excel format (blinded or unblinded as required and as per blinding status of recipient).

5.2 Statistical software

Data will be analysed using SPSS (version 28 or higher; IBM Corp., 2021) and/or Stata (version 18 or higher; StataCorp., 2023) and/or R (version 4.3.2 or higher; R Core Team, 2023).

5.3 Calculator for Index Multiple Deprivation

To measure relative deprivation, the Index of Multiple Deprivation (IMD Calculator: https://www.fscbiodiversity.uk/imd/) will be used.

5.4 Database and data entry checks

Data quality will be ensured by close monitoring and routine auditing for accuracy throughout the data collection period. In order to ensure the accuracy of the data entered into the database, 10% of key eligibility (MFQ score at baseline) and primary outcome data (MFQ score at 8 weeks following randomisation) will be checked against the raw data by someone else, blind to participant allocation, with further checks if necessary.

APPENDIX

Appendix 1: Schedule of Assessments and Measures

Trial measures

STAGES OF TRIAL	MEASURES
**Screening**	29 item MFQ - no risk items (and optional questionnaires)
**Baseline assessment before treatment**	General Information Questionnaire, MFQ, RCADS, SPPC, ISI, BADS, CRIES, AMI-Y, PIT, AMT, SCS<br>*Involvement with MH services and Risk assessment*<br>*HE measures:* CA-SUS; EQ-5D-5L; ReQoL.
**Treatment**	12 item MFQ and PHQ-9 risk item
**8, 16 and 24 week assessment**	MFQ, RCADS, SPPC, ISI, BADS, CRIES, AMI-Y, PIT, AMT, SCS<br>*Involvement with MH services and Risk assessment*<br>*HE measures:* CA-SUS; EQ-5D-5L; ReQoL.<br>*Feedback questionnaire at 8-week assessment.*

Assessments

All pupils aged 16-18 will be asked to complete screening for depression, with those scoring above cut-off (≥17 on the 29-item version of the MFQ) invited to complete the baseline assessment. Assessments will be at baseline (prior to randomisation), 8-, 16- and 24-weeks following randomisation. There is a +/-4-week visit window. Each assessment will be the same and consist of a battery of questionnaires (the majority standardized measures), the autobiographical memory task, a semi-structured clinical interview (to assess risk and history of mental health difficulties) and a semi-structured interview to measure costs for health economics (CA-SUS). Each assessment will be one hour long, and the questionnaires split into two sets: (a) essential questionnaires (general information questionnaire, MFQ, RCADS, BADS) and; (b) optional questionnaires (EQ -5D-5, ReQoL, AMI-Y, SPPC, PIT, CRIES, SCS, ISI). In the autobiographical memory task, participants are asked to provide memories to cue words (e.g., book; happy).

Appendix 2: Scoring

Mood and Feelings Questionnaire (MFQ) (Angold & Costello, 1987)

The MFQ is scored by summing together the point values of responses for each item. The response choices and their designated point values are as follows: "not true" = 0 points " sometimes true" = 1 point "true" = 2 points. No items are reversed when scoring. Higher scores on the MFQ suggest more severe depressive symptoms.

Revised Children's Anxiety and Depression Scale (RCADS-47)

The RCADS total score is scored by summing the values of responses for each item. The designated values for each item are as follows: "never" = 0 points, "sometimes" = 1 point, "often" = 2 points, "always" = 3 points. No items are reversed when scoring. Within the RCADS we score several subscales by summing the values of response for certain items. For Social Phobia subscale, sum items 4, 7, 8, 12, 20, 30, 32, 38 and 43. For Panic Disorder subscale, sum items 3, 14, 24, 26, 28, 34, 36, 39 and 41. For Separation Anxiety subscale, sum items 5, 9, 17, 18, 33, 45 and 46. For Generalised Anxiety subscale, sum items 1, 13, 22, 27, 35 and 37. For Obsessive-Compulsive subscale, sum items 10, 16, 23, 31, 42 and 44.

Behavioural Activation for Depression Scale (BADS-25) (Kanter et al., 2007)

The BADS has four subscales (activation, avoidance/rumination, school impairment and social impairment). You can also calculate a total score by summing the values of responses for each item. Respondents are provided a seven-point scale ranging from 0 (not at all) to 6 (completely). Several items on the BADS are reversed when scoring. When reversing these items, it is: 6 = 0, 5 = 1, 4 = 2, 3 = 3, 2 = 4, 1 = 5, 0 = 6). The items that need to be reversed before scoring total value are items 1, 2, 6, 8, 9, 10, 13, 14, 15, 16, 17, 18, 19, 20, 21, 22, 24 and 25.

EQ-5D-5L

Participants rate their health problems in the five dimensions 'mobility', 'self-care', 'usual activities', 'pain/discomfort' and 'anxiety/depression' on an ordinal five level scale with "no problems (1)", "slight problems (2)", "moderate problems (3)", "severe problems (4)" or "extreme problems (5)". The 5 domains will be converted into an index value as per the EQ5D-5L scoring guide by applying a formula that attaches values (weights) to each of the levels in each dimension. In addition, QoL is assessed on the visual analogue scale of the EQ-5D. Participant rate their QoL visually between 0 (worst) and 100 (best).

ReQol

The ReQol score is scored by summing the values for the responses for the ten items related to mental health. Against each response is a value ranging from 0 to 4 (some items are worded positively and some negatively). The ReQoL-10 index score can be calculated by summing the numbers for the first 10 questions on the first page. If a single question is unanswered in the first 10 questions, the mean value of the other responses can be used to fill the gap. The minimum score for ReQoL-10 is 0 and the maximum is 40, where 0 indicates poorest quality of life and 40 indicates highest quality of life. There is a single physical health question scored from zero to four.

AMI-Y

The AMI-Y has a negative imagery and positive imagery scale. The items are scored on a five-point scale from 0 to 4. Total scores are calculated by adding the scores from the items 2 and then 4 to 15. Items 1 and 3 are descriptive items. This questionnaire is being developed and evaluated during the trial.

Self-worth subscale of the Harter Self-Perception Scale (SPPC)

The SPPC total score is the sum of the values for each item response. The answers are scored on a 1 to 4 scale: where 4 represents the most adequate self-judgment and 1 represents the least adequate self-judgment. Items are counter-balanced such that item 1 and 2 are worded with the most adequate statement as "describes me very poorly" and the remaining items 3, 4, and 5 are worded with the most adequate statement as "describes me very well". Thus, items 1 and 2 are scored 4, 3, 2, 1 (from left to right), and items 3, 4, and 5 are scored 1, 2, 3, 4 (from left to right)

Future Imagery Task

The FIT has a positive and negative subscale. To score each subscale you sum the values of certain items. Values of each item are as follows: "No image at all" = 1, "Unclear and not detailed" = 2, "Unclear but some detail" = 3, "Moderately clear" = 4, "Very clear and detailed" = 5. No values are reversed. For the score of the positive subscale, you sum items 1, 3, 5, 6, 8, 9, 12 and 13. For the score of the negative subscale you sum items 2, 4, 7, 10, 11 and 14.

Child Revised Impact of Event Scale (CRIES-8)

The CRIES-8 has two subscales: Intrusion and Avoidance subscales. To score each subscale you sum the value of certain items. Values of each item are as follows: "Not at all" = 0, "Rarely" = 1, "Sometimes" = 3, "Often" = 5. No values are reversed. The score for the Intrusion subscale is the sum of items 1, 3, 6 and 7. The score for the Avoidance subscale is the sum of items 2, 4, 5 and 8.

Self-Compassion Scale – Short Form SCS

The SCS total score is scoring by summing the value for each item. Values of each item are as follows: "Never" = 1, "Rarely" = 2, "Sometimes" = 3, "Often" = 4, "Always" = 5. Certain items are reversed scored. These items are then valued as follows: "Never" = 5, "Rarely" = 4, "Sometimes" = 3, "Often" = 2, "Always" = 1. Items that are reversed scored are 1, 4, 8, 9, 11 and 12.

Insomnia Severity Index (ISI)

The total ISI score is summing the value of all seven items. No items are reverse scored. Questions 1, 2 and 3 are valued as follows: "None" = 0, "Mild" = 1, "Moderate" = 2, "Severe" = 3, "Very Severe" = 4. Question 4 is valued as follows: "Very Satisfied" = 0, "Satisfied" = 1, "Moderately Satisfied" = 2, "Dissatisfied" = 3 "Very Dissatisfied" = 4. Question 5 is valued as follows: "Not at all noticeable" = 0, "A little" = 1, "Somewhat" = 2, "Much" = 3, "Very much noticeable" = 4. Question 6 is valued as follows: "Not at all worried" = 0, "A little" = 1, "Somewhat" = 2, "Much" = 3, "Very much worried" = 4. Question 7 is valued as follows: "Not at all interfering" = 0, "A little" = 1, "Somewhat" = 2, "Much" = 3, "Very much interfering" = 4.

Disturbing Dream and Nightmare Severity Index

The Disturbing Dream and Nightmare Severity Index (DDNSI) is a 5-item self-report scale assessing nightmare severity. Questions relate to nights per week with nightmares, nightmare frequency, awakenings, severity of nightmare problem, and the intensity of nightmares. The total score ranges from 0 to 37, with higher scores indicating a more severe problem. To score, add nights/per week (0 to 7) + nightmares/week (maximum for nightmares/week = 14, so scale is 0 to 14) + Q3 (0 to 4 scale) +Q4 (0 to 6 scale) + Q5 (0 to 6 scale). Q2 is not included in the total score.

REFERENCES

Angold, A., Costello, E. J., Messer, S. C., Pickles, A., Winder, F., & Silver, D. (1995). The development of a short questionnaire for use in epidemiological studies of depression in children and adolescents. *International Journal of Methods in Psychiatric Research*, *5*, 237–249.

Bastien, H., Vallieres, A., & Morin, C. M. (2001). *Validation of the Insomnia Severity Index as an outcome measure for insomnia research*. www.elsevier.com/locate/sleep

Burleson Daviss, W., Birmaher, B., Melhem, N. A., Axelson, D. A., Michaels, S. M., & Brent, D. A. (2006). Criterion validity of the Mood and Feelings Questionnaire for depressive episodes in clinic and non-clinic subjects. *Journal of Child Psychology and Psychiatry and Allied Disciplines*, *47*(9), 927–934. https://doi.org/10.1111/j.1469-7610.2006.01646.x

Chorpita, B. F., Moffitt, C. E., & Gray, J. (2005). Psychometric properties of the Revised Child Anxiety and Depression Scale in a clinical sample. *Behaviour Research and Therapy*, *43*(3), 309–322. https://doi.org/10.1016/j.brat.2004.02.004

Costello, E. A. A. (1988). Scales to assess child and adolescent depression: checklists, screens, and nets. . *J Am Acad Child Adolesc Psychiatry*, *6*, 726–737.

Devilly, G. J., & Borkovec, T. D. (2000). Psychometric properties of the credibility/ expectancy questionnaire. In *Journal of Behavior Therapy and Experimental Psychiatry* (Vol. 31).

Eg, J., Bilenberg, N., Costello, E. J., & Wesselhoeft, R. (2018). Self- and parent-reported depressive symptoms rated by the mood and feelings questionnaire. *Psychiatry Research*, *268*, 419–425. https://doi.org/10.1016/j.psychres.2018.07.016

Freeman, D., Emsley, R., Diamond, R., Collett, N., Bold, E., Chadwick, E., Isham, L., Bird, J. C., Edwards, D., Kingdon, D., Fitzpatrick, R., Kabir, T., Waite, F., Carr, L., Causier, C., Černis, E., Kirkham, M., Lambe, S., Lister, R., … Twivy, E. (2021). Comparison of a theoretically driven cognitive therapy (the Feeling Safe Programme) with befriending for the treatment of persistent persecutory delusions: a parallel, single-blind, randomised controlled trial. *The Lancet Psychiatry*, *8*(8), 696–707. https://doi.org/10.1016/S2215-0366(21)00158-9

Hackmann, A., Clark, D. M., & McManus, F. (2000). Recurrent images and early memories in social phobia. *Behaviour Research and Therapy*, *38*(6), 601–610.

Harter S. (1999). *The construction of the self: a developmental perspective. 1st ed. New York: Guilford Press*.

Harter, S. (2012). *The construction of the self: Developmental and socio-cultural foundations.* Guilford Press.

Herdman, M., Gudex, C., Lloyd, A., Janssen, M., Kind, P., Parkin, D., Bonsel, G., & Badia, X. (2011). Development and preliminary testing of the new five-level version of EQ-5D (EQ-5D-5L). *Quality of Life Research*, *20*(10), 1727–1736. https://doi.org/10.1007/s11136-011-9903-x

Holmes, E. A., Lang, T. J., Moulds, M. L., & Steele, A. M. (2008). Prospective and positive mental imagery deficits in dysphoria. *Behaviour Research and Therapy*, *46*(8), 976–981. https://doi.org/10.1016/j.brat.2008.04.009

IBM Corp. (2021). *IBM SPSS Statistics for Windows, Version 28.0.* . Armonk, NY: IBM Corp.

Kanter, J. W., Mulick, P. S., Busch, A. M., Berlin, K. S., & Martell, C. R. (2007). The Behavioral Activation for Depression Scale (BADS): Psychometric properties and factor structure. *Journal of Psychopathology and Behavioral Assessment*, *29*(3), 191–202. https://doi.org/10.1007/s10862-006-9038-5

Keetharuth, A. D., Brazier, J., Connell, J., Bjorner, J. B., Carlton, J., Buck, E. T., Ricketts, T., McKendrick, K., Browne, J., Croudace, T., Barkham, M., Blenkiron, P., Boardman, J., Everett, S. H., Grundy, A., Hanlon, R., Hemmingfield, J., Papadopoulos, A., Robotham, D., … Slade, M. (2018). Recovering Quality of Life (ReQoL): A new generic self-reported outcome measure for use with people experiencing mental health difficulties. *British Journal of Psychiatry*, *212*(1), 42–49. https://doi.org/10.1192/bjp.2017.10

Neff, K. D. (2003). The Development and Validation of a Scale to Measure Self-Compassion. *Self and Identity*, *2*(3), 223–250. https://doi.org/10.1080/15298860309027

Perrin, S., Meiser-Stedman, R., & Smith, P. (2005). The Children's Revised Impact of Event Scale (CRIES): Validity as a Screening Instrument for PTSD. *Behavioural and Cognitive Psychotherapy*, *33*(04), 487. https://doi.org/10.1017/S1352465805002419

Pile, V., & Lau, J. Y. F. (2018). Looking Forward to the Future: Impoverished Vividness for Positive Prospective Events Characterises Low Mood in Adolescence. *Journal of Affective Disorders*, *238*, 269–276. https://doi.org/10.1016/j.jad.2018.05.032

Pile, V., Schlepper, L. K., Lau, J. Y. F., & Leamy, M. (2023). An early intervention for adolescent depression targeting emotional mental images and memory specificity: a process evaluation. *European Child and Adolescent Psychiatry*, *32*(5), 783–795. https://doi.org/10.1007/s00787-021-01902-7

Pile, V., Smith, P., Leamy, M., Oliver, A., Bennett, E., Blackwell, S. E., Meiser-Stedman, R., Stringer, D., Dunn, B. D., Holmes, E. A., & Lau, J. Y. F. (2021). A feasibility randomised controlled trial of a brief early intervention for adolescent depression that targets emotional mental images and memory specificity (IMAGINE). *Behaviour Research and Therapy*, *143*, 103876. https://doi.org/10.1016/j.brat.2021.103876

R Core Team. (2023). *R: A Language and Environment for Statistical Computing.* R Foundation for Statistical Computing.

Raes, F., Pommier, E., Neff, K. D., & Van Gucht, D. (2011). Construction and factorial validation of a short form of the Self-Compassion Scale. *Clinical Psychology and Psychotherapy*, *18*(3), 250–255. https://doi.org/10.1002/cpp.702

Short, M. A., Gradisar, M., Lack, L. C., Wright, H. R., & Chatburn, A. (2013). Estimating adolescent sleep patterns: Parent reports versus adolescent self-report surveys, sleep diaries, and actigraphy. *Nature and Science of Sleep*, *5*, 23–26. https://doi.org/10.2147/NSS.S38369

StataCorp. (2023). *Stata Statistical Software: Release 18.* College Station, TX: StataCorp LLC. .

Stober, J. (2000). Prospective cognitions in anxiety and depression: Replication and methodological extension. *Cognition and Emotion*, *9931*, 37–41. https://doi.org/10.1080/02699930050117693

Thabrew, H., Stasiak, K., Bavin, L. M., Frampton, C., & Merry, S. (2018). Validation of the Mood and Feelings Questionnaire (MFQ) and Short Mood and Feelings Questionnaire (SMFQ) in New Zealand help-seeking adolescents. *International Journal of Methods in Psychiatric Research*, *27*(3). https://doi.org/10.1002/mpr.1610

Vallis, T. M., Shaw, B. F., & Dobson, K. S. (1986). The Cognitive Therapy Scale: Psychometric properties. *Journal of Consulting and Clinical Psychology*, *54*(3), 381–385. https://doi.org/10.1037/0022-006X.54.3.381

Williams, J. M. G., & Broadbent, K. (1986). Autobiographical memory in suicide attempters. *Journal of Abnormal Psychology*, *95*(2), 144–149. https://doi.org/10.1037/0021-843X.95.2.144
